# EST‐SSR‐based landscape genetics of *Pseudotaxus chienii*, a tertiary relict conifer endemic to China

**DOI:** 10.1002/ece3.7769

**Published:** 2021-06-15

**Authors:** Shufeng Li, Zhen Wang, Yingjuan Su, Ting Wang

**Affiliations:** ^1^ School of Life Sciences Sun Yat‐sen University Guangzhou China; ^2^ Research Institute of Sun Yat‐sen University in Shenzhen Shenzhen China; ^3^ College of Life Sciences South China Agricultural University Guangzhou China

**Keywords:** adaptive evolution, EST‐SSR, genetic differentiation, genetic diversity, landscape genetics, *Pseudotaxus chienii*

## Abstract

*Pseudotaxus chienii*, belonging to the monotypic genus *Pseudotaxus* (Taxaceae), is a relict conifer endemic to China. Its populations are usually small and patchily distributed, having a low capacity of natural regeneration. To gain a clearer understanding of how landscape variables affect the local adaptation of *P*. *chienii*, we applied EST‐SSR markers in conjunction with landscape genetics methods: (a) to examine the population genetic pattern and spatial genetic structure; (b) to perform genome scan and selection scan to identify outlier loci and the associated landscape variables; and (c) to model the ecological niche under climate change. As a result, *P*. *chienii* was found to have a moderate level of genetic variation and a high level of genetic differentiation. Its populations displayed a significant positive relationship between the genetic and geographical distance (i.e., “isolation by distance” pattern) and a strong fine‐scale spatial genetic structure within 2 km. A putatively adaptive locus EMS6 (functionally annotated to cellulose synthase A catalytic subunit 7) was identified, which was found significantly associated with soil Cu, K, and Pb content and the combined effects of temperature and precipitation. Moreover, *P*. *chienii* was predicted to experience significant range contractions in future climate change scenarios. Our results highlight the potential of specific soil metal content and climate variables as the driving force of adaptive genetic differentiation in *P*. *chienii*. The data would also be useful to develop a conservation action plan for *P*. *chienii*.

## Graphical abstract

Landscape and environmental heterogeneity play important roles in the spatial genetic structure and the adaptive evolution of *P*. *chienii*. Adaptive gene indeed occurred in genome of *P*. *chienii* and made the plant to have adaptive potential in response to severe environmental stress. These factors related to adaptation should be specially considered in ex situ conservation of *P*. *chienii*.

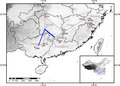

## 1 INTRODUCTION

Several spatial changes related to geography or environment, such as isolation, fragmentation or spatial reduction, may have profound demographic and negative genetic consequences for species. Landscape features, range boundaries, or environmental characteristics are well known to influence both population genetic differentiation and spatial genetic structure. The theory of isolation by distance (IBD) (Wright, 1943) expects that genetic differentiation increases with geographical distance, while that of isolation by environment (IBE) concerns that genetic differentiation increases with environmental differences, independent of geographical distance (Orsini et al., 2013; Wang & Bradburd, 2014). Extensive research finds that a substantial number of species have independently or jointly experienced IBD and IBE patterns (Sexton et al., 2014). Hence, investigation on adaptive genetic variation of populations and their adaptability to environmental change are essential to forecast the persistence of endemic endangered plants in future climates.

Landscape genetics is one of the most promising approaches to explore how landscape pattern, structure, and composition affect spatial genetic variation of populations, continuity of gene flow, and local adaptation (Balkenhol et al., 2016; Manel et al., 2003). It can determine and quantify the relationship between complex and dynamic landscape and various genetic evolutionary processes (Storfer et al., 2007). Effects of geographical distance or environmental configuration on among‐population gene flow and genetic differentiation (e.g., IBD or IBE) have been revealed by using landscape genetics approaches (Chau et al., 2019; González‐Martínez et al., 2010; Hübner et al., 2009; Tóth et al., 2019). Importantly, adaptive genetic differentiation and local adaptation processes are found to be possibly associated with multiple environmental variables (Hancock et al., 2011; Manel et al., 2012; Mosca et al., 2014; Pal et al., 2020; Shih et al., 2018). And candidate adaptive loci may function in growth, phenology, or stress resistance (Eckert et al., 2010; Namroud et al., 2008; Shih et al., 2018; Song et al., 2016).

Several genome scan methods have been developed to detect signatures of selection. By using *F*_ST_‐based tests, outliers can be detected in genomic regions potentially under selection through comparing genetic differentiation at given loci with a neutral baseline distribution (Luikart et al., 2003). However, the major drawback of this method is the existence of false positives derived from null alleles, complex population genetic structure, and demographic history such as bottlenecks and allele surfing (Bierne et al., 2011; Foll & Gaggiotti, 2008; Jones et al., 2013; Strasburg et al., 2012). It is also difficult for this method to capture weak divergent selection (De Villemereuil et al., 2014; Narum & Hess, 2011) and other methods to detect selection by correlating genetic variation with environmental variables. They have the advantage to increase the probability of detecting weak selection and to provide evidence of adaptation to environmental change in association with functional genes (De Mita et al., 2013). Nevertheless, factors like pre‐existing population genetic structure (Novembre & Di Rienzo, 2009) and correlations among environmental variables (De Villemereuil et al., 2014) may lead to false adaptive candidate loci. It is noteworthy that environmental selection requires enough time to trigger a change in the pattern of allele frequency differentiation (Joost et al., 2013), and neutral demography or migration history may also generate an environmental pattern that is irrelative to adaptation (Novembre & Di Rienzo, 2009). To ensure power and accuracy, researchers tend to simultaneously adopt more than two approaches to identify robust outlier candidates (Pal et al., 2020; Shih et al., 2018; Song et al., 2016).

Ecological niche modeling (ENM) has been widely applied: (a) to predict species distributions (Dakhil et al., 2019; Gilani et al., 2020), (b) to identify climate refugia (Leite et al., 2016; Liu et al., 2013), (c) to determine the impact of invasive species (Banerjee et al., 2019; Padalia et al., 2014), and (d) to evaluate the effects of climate change on species (Shao et al., 2017; Yan et al., 2017). Notably, it is reasonable to integrate ENM within the landscape genetics framework, because the latter has the potential to identify environmental variables associated with adaptive genetic variation. As for endangered plants, the integration may facilitate the prediction of suitable ranges under climatic change and inform conservation measures. For instance, a combination of the two methods has been used to modeling the climatically suitable areas of *Pinus bungeana* (Zhang et al., 2019).

**FIGURE 1:**
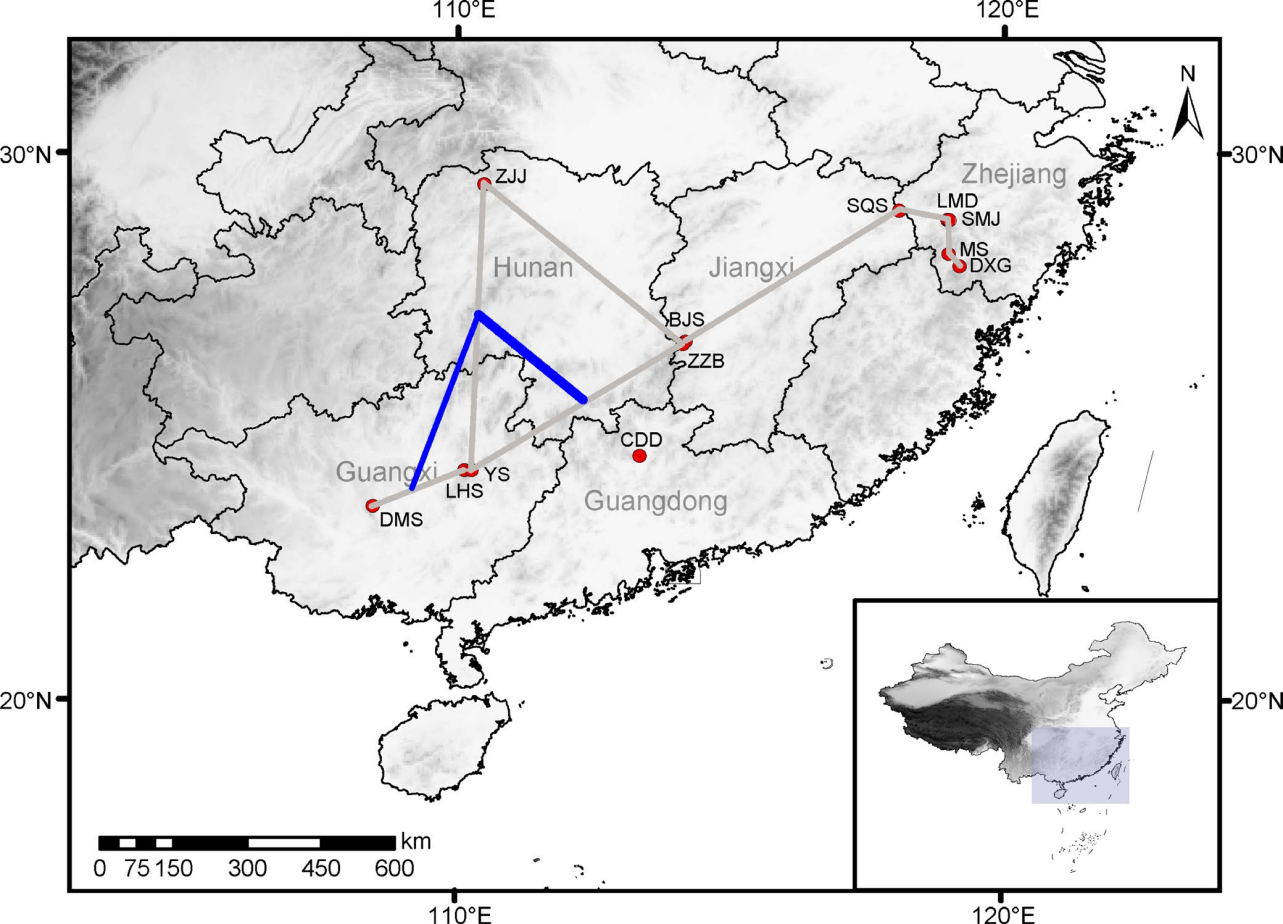
Sampling locations of 11 *Pseudotaxus chienii* populations and genetic boundaries (blue lines) identified by Monmonier's algorithm. The width of blue lines represents the “strength” of the boundaries

*Pseudotaxus chienii* is a relict endangered conifer endemic to China, belonging to the monotypic genus *Pseudotaxus* (Fu et al., 1999; Kou et al., 2020). The species is a dioecious woody shrub or small tree up to 4 m tall (Fu et al., 1999). Its seeds are partly enclosed within a fleshy white aril at maturity, which may be dispersed by birds or small animals (Fu et al., 1999; Wang et al., 2006). Natural populations of *P*. *chienii* occur in montane regions of southern Zhejiang, southwestern Jiangxi, northwestern and southern Hunan, northern Guangxi, and northern Guangdong, China (Figure 1; Fu et al., 1999). They are usually small and isolated, thought to have long been patchily distributed (Fu & Jin, 1992). *Pseudotaxus chienii* plants primarily grow in the understory of evergreen and deciduous broad‐leaved forests at altitudes of 700–1,500 m. They are usually found on sites with acidic (pH 4.2–4.5) and nutrient‐rich soils, receiving an annual mean precipitation of 1,800–2,400 mm (Fu & Jin, 1992). *Pseudotaxus chienii* has undergone a population reduction of more than 30% over the past decades due to overexploitation and habitat loss (Su et al., 2009; Thomas & Yang, 2013), which is further aggravated by difficulty of reproduction and seedling establishment. Currently, *P*. *chienii* has been categorized as an endangered species in the Red List of Endangered Plants in China (Fu & Jin, 1992) and as a vulnerable (VU) species by the International Union for Conservation of Nature (IUCN) (Thomas & Yang, 2013).

Previous investigations show that *P*. *chienii* has low genetic diversity and high genetic differentiation (Su et al., 2009; Wang et al., 2006; Zhou et al., 1998). However, its adaptive adaptation to environment remains unclear. In particular, little is known about its genes or genomic regions under selection, which is essential for formalizing the conservation of *P*. *chienii* in complex heterogeneous landscapes like mountain ecosystems. Moreover, the knowledge is also a prerequisite for a successful migration of *P*. *chienii* from adversely environmental stresses. Of note, expressed sequence tag‐simple sequence repeats (EST‐SSRs) have been widely applied to examine adaptive genetic variation and local adaptation in both model and nonmodel species (Alcaide et al., 2019; Bradbury et al., 2013; Lind‐Riehl et al., 2014; Saini et al., 2019).

In this study, we used EST‐SSRs in junction with landscape genetics statistical methods to explore the possible roles of geographical location and environmental factors played in shaping the population genetic variation of *P*. *chienii*. Our specific aims were to (a) characterize the level and pattern of genetic variation, genetic differentiation, and spatial genetic structure of *P*. *chienii* populations across its entire distribution range, (b) identify candidate outlier loci as well as their association with environmental variables, and (c) model the distribution of *P*. *chienii* under future climate changes and estimate the major factors affecting the distribution. These investigations may provide new information to deepen our understanding of the adaptation of *P*. *chienii* populations and assist the development of conservation strategies.

## 2 MATERIALS AND METHODS

### 2.1 Sample collection and DNA extraction

A total of 134 *P*. *chienii* individuals were collected from 11 populations in Zhejiang, Guangxi, Jiangxi, and Hunan provinces (Figure 1; Table S1), which covered its whole distribution in China. Fresh leaves were sampled randomly with 19 to 31 individuals for each population. The sampling interval was at least 30 m. Leaves were dried with silica gel and stored at −20°C until DNA extraction. Geographical and altitude information were acquired with GPS (Table S1).

Total genomic DNA was extracted using a modified cetyltrimethylammonium bromide (CTAB) protocol (Su et al., 1998). Its quality and quantity were measured by 0.8% (*w/v*) agarose gel electrophoresis and a NanoDrop 2000c spectrophotometer (Thermo Fisher Scientific, Waltham, MA, USA). DNA was diluted to 50 ng/μl and stored at −20°C for subsequent use.

### 2.2 Genotyping using EST‐SSR markers

All individuals of *P*. *chienii* were genotyped using 20 polymorphic EST‐SSR markers previously developed by Xu et al. (2020) (Table S2). PCRs were performed in 25 μl volume containing 1 μl template DNA (50 ng/μl), 2.5 μl 10× PCR Buffer (with Mg^2+^), 1.6 μl dNTPs (2.5 mM), 0.5 μl of each forward and reverse primers (10 μM), and 0.2 μl Taq DNA polymerase (5 U/μl) (Takara, Dalian, China). Negative control was set without genomic DNA. All forward primers were labeled with fluorescent dyes 5‐FAM (Invitrogen, Shanghai, China). PCR amplifications were performed in a thermal cycler (Veriti, Applied Biosystems, Foster City, CA, USA) as follows: initial denaturation at 94°C for 5 min; followed by 35 cycles at 94°C for 40 s, varied annealing temperatures ranging from 55 to 62°C for 40 s with different primer pairs (Table S2), and extension at 72°C for 30 s; and a final extension at 72°C for 10 min.

Subsequent genotyping was performed by using capillary gel electrophoresis on an ABI 3730xl automated Genetic Analyzer (Applied Biosystems). Allele sizes were determined with GeneMapper 4.0 software (Applied Biosystems). Each genotype was visually checked and scored, and unclear samples were re‐amplified. We randomly selected two individuals per population to test reproducibility through two random primers. Genotyping error rate was detected as 5.3% using the sample function in R (R Core Team, 2013).

### 2.3 Population genetic diversity and differentiation

Micro‐Checker version 2.2.3 (Van Oosterhout et al., 2004) was used to check null alleles based on 1,000 Monte Carlo simulation. The *basicStats* function and *divBasic* function of R package diveRsity1.9.90 (Keenan et al., 2013) were used to assess genetic parameters, including the number of different alleles (*A*), allelic richness (*Ar*), observed heterozygosity (*Ho*), expected heterozygosity (*He*), unbiased expected heterozygosity (*uHe*), inbreeding coefficient (*F*_IS_), and fixation index (*F*). Allele frequency was calculated by the *makefreq* function of R package adegenet 2.1.1 (Jombart, 2008). Private alleles were estimated using the *private_alleles* function of R package poppr 2.8.3 (Kamvar et al., 2014). We used the *basic.stats* function of R package hierfstat 0.04‐22 (Goudet, 2005) to calculate observed heterozygosity (*Ho*), gene diversity within population (*Hs*) and overall gene diversity (*Ht*). Multilocus linkage disequilibrium (LD) was assessed by index of association (*I*_a_) (Brown et al., 1980) and standardized index of association (*r*_D_) (Agapow & Burt, 2001). *I*_a_ and *r*_D_ of pairwise locus, each population, and all populations were calculated using the *pair.ia*, *poppr*, and *ia* functions in the R poppr package with 999 permutations, respectively.

The departure from Hardy–Weinberg equilibrium for the loci was tested using *test_HW* function in the R package genepop 1.1.2 (Rousset, 2008), with the Markov chain parameters set at 10,000 dememorization steps, 20 batches, and 5,000 iterations per batch.

Linear mixed effect model (LMM) with reduced maximum‐likelihood estimation was used to assess the difference of mean *uHe* per locus at the population and province levels using the *lmer* function in the R package lme4 1.1‐21 (Bates et al., 2015). In LMM, population or province was treated as a fixed effect, whereas locus as a random effect. A likelihood‐ratio test using the ANOVA function in the R package car (Fox & Weisberg, 2011) was carried out to test the overall difference at the population and province levels. Tukey's HSD post hoc comparison was further conducted using the *glht* function in the R package multcomp 1.4‐10 (Hothorn et al., 2008).

Due to the difference in population size, we assessed the correlations between genetic parameters (*Ar*, *Ho*, *He*, *uHe*, and *F*) and population size (*Ns*) using the *corr.tes* function in the R package psych 1.8.12 (Revelle, 2018). Effects of population size on genetic diversity and differentiation and the association between variables were quantified by Pearson's correlation coefficient with the Holm method to adjust the *p*‐value.

To investigate genetic differentiation, *F*‐statistics (Weir & Cockerham, 1984) for each locus and pairwise *F*_ST_ at the population, province, and species levels were evaluated using the *diffCalc* function in the R package diveRsity, with 95% confidence intervals (CI) and 1,000 bootstrap replicates. Analysis of molecular variance (AMOVA) was performed using the *poppr.amova* function in the R package poppr. And the following Φ indices were estimated: within individuals, Φ_IT_; among individuals within populations, Φ_IS_; and among populations, Φ_ST_. The *randtest* function was used to assess the significance of the Φ indices.

Nei's distance (Nei, 1972, 1978) was calculated using the *dist.genpop* function in the R package adegenet. A heatmap with UPGMA clustering was constructed using the *hclust* and the *heatmap.2* functions of the R package gplots 3.0.1.1 (Warnes et al., 2016).

### 2.4 Population genetic structure

A Bayesian clustering approach was performed using STRUCTURE 2.3.4 (Falush et al., 2003, 2007; Pritchard et al., 2000) to determine the number of genetically homogeneous groups of individuals and to assess the amount of admixture between individuals with the admixture model and allele frequencies correlated. We ran the program with 100,000 burn‐in, 100,000 Markov Chain Monte Carlo (MCMC) iterations, putative *K* ranging from 1 to 14, and 20 replicated runs. Based on the highest Delta‐*K* value (Evanno et al., 2005), the optimal K was determined through an online program STRUCTURE HARVESTER (Earl & vonHoldt, 2012). *K* values were summarized using CLUMPP 1.1.2.b (Jakobsson & Rosenberg, 2007) to obtain the cluster membership coefficient of each population (Q‐matrix), and the final output was visualized using Distruct v 1.1 (Rosenberg, 2004).

In order to estimate population divergence, we used the *find.clusters* function of the R package adegenet to conduct principal component analysis (PCA) and define *k* clusters using the *K*‐means clustering algorithm. Bayesian information criterion (BIC) value was used to determine the optimal cluster *k*. As a more powerful method, discriminant analysis of principal components (DAPC) (Jombart et al., 2010) was also conducted using the same function in R as PCA. To control for possible overfit, we used cross‐validation to determine the best PC numbers through the *xvalDapc* function in the same package with 90% data as training set and the remaining 10% as validation set. As a result, 40 PCs were used in DAPC analysis.

### 2.5 Landscape variable dataset

Based on field survey, published and online data, we constructed a landscape variable dataset for *P*. *chienii* populations, which included geographical and environmental variables. The former contained longitude and latitude, whereas the latter included six ecological, 19 bioclimate, and 20 soil variables (Appendix S2).

### 2.6 Landscape heterogeneity test

Population landscape variables were regarded as variables for its all individuals. Based on these variables of individuals, we performed permutational multivariate analysis of variance (PERMANOVA) to test landscape heterogeneity among 11 populations and four provinces using the *adonis* function of the R package vegan 2.5‐5 (Dixon, 2003). Euclidean distance matrices were generated as response variables for PERMANOVA with 999 permutations. Using the same permutations, we also conducted pairwise comparisons between populations or provinces through the *pairwise.perm*.*MANOVA* function of the R package RVAideMemoire 0.9‐73 (Hervé, 2018).

### 2.7 Isolation pattern detection

We used three strategies to evaluate isolation by distance (IBD) and isolation by environment (IBE) for *P*. *chienii* populations. The geographical and environmental variables with variance inflation factor (VIF) below 5 were selected for analysis in the *vifstep* function of the R package *usdm* (Naimi et al., 2014). VIF was used to measure the correlation between two or more predictor variables (collinearity). The larger the VIF, the stronger linear relationship of the variables with at least one of the other variables. Before formal analysis, the selected geographical or environmental variables were scaled in the *scale* function, and the Euclidean geographical or environmental distance was subsequently calculated in the *dist* function.

Firstly, we conducted Mantel test to evaluate the relationship between genetic distance (pairwise *F*_ST_) and Euclidean geographical or environmental distance using the *mantel* function in the R package *vegan* with 10,000 permutations. Secondly, a partial Mantel test was further used to distinguish which geographical or environmental variables may have affected genetic distance through controlling one of the two types of variables using the *mantel.partial* function of the R package vegan with 10,000 permutations. In the two tests, the association between variables was quantified by Pearson's correlation coefficient. Finally, we applied a multiple matrix regression with randomization analysis (MMRR) to investigate the effects of geographical and environmental distance on genetic distance in the R script *MMRR* (deposited in the Dryad Data Repository under https://doi.org/10.5061/dryad.kt71r) with 999 permutations (Wang, 2013).

### 2.8 Effects of geography and environment on genetic variation

To quantify the contribution of IBD and IBE to the genetic differentiation of *P*. *chienii* populations, we conducted redundancy analysis (RDA) using the *varpart* function of the R package *vegan*. Hellinger transformation was used to transform genetic data into response variables of RDA models using the *decostand* function in R. Predictor variables included the geographical and environmental variables with VIF below 5. We used the *anova*.*cca* and *rda* functions to estimate the contribution of a single and all predictor variables to the genetic variation with 999 permutations.

Associations between outliers and landscape variables were assessed using two methods: Samβada v.0.8.1 (Stucki et al., 2017) and linear mixed‐effects model (LMM). The latter was performed using the *lmer* function of the R package *lmer4*. Two geographical variables and 16 environmental variables with VIF < 5 were used for environmental association analysis. To preserve the diversity of environmental factors as much as possible, we applied the *vifstep* function for three categories of environmental variables. Sixteen selected environmental variables included five ecological variables (altitude; percent tree cover, percent tree cover (PTC); enhanced vegetation index, EVI; leaf area index, LAI; and fraction of absorbed photosynthetically active radiation, fPAR), four bioclimate variables (Bio10, Bio11, Bio13, and Bio14), and seven soil variables (K, Na, Fe, Mn, Zn, Cu, and Pb). A multiple univariate logistic regression approach was employed to test correlations between allele frequencies and environmental variables. We compared models with and without environmental variables, and the significance was determined based on both Wald and G scores with a false discovery rate (FDR) cutoff of 10^–6^. As for the allele frequencies of outliers, LMM was constructed using the *lmer* function with landscape variables as the fixed effect and provinces as the random effect. The significance of difference was determined through a likelihood‐ratio test using the ANOVA function in R.

### 2.9 Investigation of spatial genetic structure

We assessed the fine‐scale spatial genetic structure (FSGS) using SPAGeDi v1.3 (Hardy & Vekemans, 2002). Generally, genetic differentiation is expected to increase with the spatial distance under limited dispersal (Vekemans & Hardy, 2004). Kinship coefficients (*F*_ij_) (Loiselle et al., 1995) between pairwise individuals were calculated at six distance intervals: 0–2 km, 2–4 km, 4–6 km, 6–8 km, 8–10 km, and 10–12 km. The regression slope (*b*_Ld_) was acquired through *F*_ij_ regressing on the natural logarithm of the spatial distance (ln(*d*_ij_)). The *Sp* values were calculated using *Sp* = b/(*F*_1_−1), based on the *F*_ij_ of the first distance class, to quantify the strength of the fine‐scale spatial genetic structure (Vekemans & Hardy, 2004).

We also used software SAM v4.0 to determine the spatial autocorrelation at the large scale, whose strength was further quantified using Moran's *I* statistic based on geographical coordinates and *uHe* of each population (Rangel et al., 2010). Moran's *I* was estimated at eight distance intervals with 9,999 permutations.

### 2.10 Demographic history

BOTTLENECK 1.2.02 (Piry et al., 1999) was used to test bottleneck effect through assessment of heterozygosity excess, which correlated the expected heterozygosity (*He*) and observed heterozygosity (*Ho*) at mutation‐drift equilibrium. The analysis was conducted under two mutation models: the stepwise mutation model (SMM) and the two‐phase mutation model (TPM) which were suitable for microsatellite data (Di Rienzo et al., 1994; Piry et al., 1999). Wilcoxon sign‐rank test was used to obtain the statistical significance with 1,000 simulations. In addition, the “mode‐shift” of allele frequency distribution was applied to distinguish bottlenecked populations (Luikart et al., 1998).

In view of the heterogeneity of *P*. *chienii* distribution, we employed Monmonier's maximum difference algorithm (Manni et al., 2004; Monmonier, 1973) to assess its genetic discontinuities based on the Euclidean distance of genetic dataset and geographical coordinates of populations, using the *monmonier* function of the R adegenet package. The Gabriel graph was used to construct connection network for *P*. *chienii* populations using the *chooseCN* function. To reduce noise, we performed a principal component analysis (PCA) for the Euclidean distance of genetic data using the *dudi.pco* function in the R ade4 1.7‐13 package (Dray & Dufour, 2007), whose first eigenvalue was further adopted for the Monmonier algorithm. The default threshold (d, third quartile of all the distances between neighbors) was used in the *monmonier* function. Assessments of migration level between populations and the construction of a weighted network were obtained using the *divMigrate* function of the R package *diveRsity* (Sundqvist et al., 2016). Only relative migration values over 0.1 were considered, which was obtained using the *Nm* statistic with 1,000 replicates.

### 2.11 Test for *F*_ST_ outliers

BAYESCAN (Foll & Gaggiotti, 2008) and FDIST (Beaumont & Nichols, 1996) were used to identify *F*_ST_ outliers (Foll & Gaggiotti, 2008). BAYESCAN implements reversible jump Markov chain Monte Carlo algorithm to estimate the ratio of posterior probabilities of selection over neutrality, namely the posterior odds (PO). In this study, we used 10 pilot runs of 5,000 iterations and a sample size of 50,000 with a thinning interval of 20. Only loci with log_10_PO > 0.5 were considered as outliers, which could be visualized by using the *plot_bayescan* function in R. FDIST detected outliers by the comparison of observed *F*_ST_ and *uHe* to those derived from simulated neutral distributions under a 99.5% confidence interval (CI) and 1% FDR. Parameters were set as follows: critical frequency 0.99, level of differentiation (target average θ) 0.06, 5,000 resamplings, Zhivotovsky parameters 0.25, trimmed mean P 0.3, and smoothing proportion 0.04.

### 2.12 Construction of ecological niche modeling

We employed a maximum entropy model in MaxEnt 3.4.1 (Phillips et al., 2006) to simulate the distribution of *P*. *chienii* under current (1950–2000), near‐future at 2050 (2041–2060), and far‐future at 2070 (2061–2080) periods. Species occurrence records were collected from the fieldwork, literature, the Global Biodiversity Information Facility (GBIF, https://www.gbif.org/), and Chinese Virtual Herbarium (CVH, http://www.cvh.ac.cn/). In total, 51 occurrence points were obtained after removing duplicate geographical records.

The climatic layers of 19 bioclimatic variables under current and future periods were downloaded from the WorldClim database (http://www.worldclim.org/) with a resolution of 2.5 arc‐minutes. We predicted future distributions based on four RCPs (representative concentration pathways) scenarios from the Community Climate System Model (CCSM4): RCP2.6, RCP4.5, RCP6.0, and RCP8.5 scenarios. RCP4.5 and RCP6.0 scenarios exhibit stable scenarios for the greenhouse gas emission, whereas RCP2.6 and RCP8.5 represent lower and higher greenhouse gas emission, respectively (Moss et al., 2010; Van Vuuren et al., 2011). To avoid redundancy, variables with VIF ≥5 were removed. Five variables (Bio2, Bio7, Bio10, Bio14, and Bio18) were used in the MaxEnt with 10 cross‐validation replicates for each model of three periods. We estimated the contribution of environmental variables to the *P*. *chienii* distribution using Jackknife test in MaxEnt (Elith et al., 2006). The area under the receiver operating characteristic curve (AUC) (Phillips et al., 2006) was used to evaluate the performance of the models.

## 3 RESULTS

### 3.1 Genetic diversity within populations

Based on 20 polymorphic EST‐SSR markers, we obtained 164 alleles with an average of 8.2 alleles per locus. Except for loci EMS1, EMS3, EMS4, EMS16, EMS18, and EMS20, the other loci were found to have null alleles in *P*. *chienii* populations (Table S3). The null allele frequencies varied from 0.0951 to 0.4142, with the highest value in DMS for locus EMS15. A significant departure from Hardy–Weinberg equilibrium was detected in majority of the populations for 20 EST‐SSR loci (Table S4).

Allelic richness in each population ranged from 2.329 (LHS) to 3.406 (LMD), with an average of 2.868 (Table 1). We identified a total of 49 private alleles in populations. Compared to population MS (the number of samples, Ns = 31; the number of private alleles, Np = 3) and population LMD (Ns = 31, Np = 7), population ZJJ displayed disproportionately many private alleles in relation to population size (Ns = 19, Np = 10), while populations SMJ, LHS, and ZZB had the lowest private alleles (Ns = 30, Np = 2). Observed heterozygosity (mean *Ho* = 0.341) was lower than expected heterozygosity (mean *He* = 0.370) across populations. The mean inbreeding coefficient (*F*_IS_ = 0.076) of each population indicated slight homozygote excess. Except for populations LHS and YS, the other populations exhibited a heterozygote deficit based on *F*_IS_ (Table 1).

**TABLE 1:** Genetic parameters based on 20 EST‐SSR markers of 11 *Pseudotaxus chienii* populations

Pop	*Ns*	*A*	*Ar*	*Np*	*Ho*	*He*	*uHe*	*F*_IS_	*F*	*I*_a_	*r*_D_
MS	31	67	2.878	3	0.382	0.386	0.392	0.009	0.038	0.056	0.004
DXG	23	65	2.885	5	0.315	0.343	0.351	0.082	0.138	−0.088	−0.006
LMD	31	80	3.406	7	0.352	0.404	0.410	0.129	0.180	0.017	0.001
SMJ	30	69	3.033	2	0.380	0.407	0.414	0.067	0.104	−0.170	−0.010
LHS	30	52	2.329	2	0.277	0.271	0.276	−0.021	0.029	0.158	0.012
YS	30	68	2.910	6	0.358	0.354	0.360	−0.012	0.101	0.158	0.010
DMS	30	61	2.795	5	0.372	0.393	0.400	0.055	0.158	0.223*	0.016*
BJS	30	73	3.010	3	0.350	0.381	0.388	0.082	0.309	−0.078	−0.004
ZZB	30	60	2.704	2	0.333	0.399	0.406	0.167	0.281	0.132	0.009
SQS	30	69	3.020	4	0.358	0.390	0.397	0.081	0.098	0.097	0.006
ZJJ	19	57	2.570	10	0.272	0.340	0.349	0.199	0.294	0.336*	0.023*
Mean	—	65.545	2.868	4.455	0.341	0.370	0.377	0.076	0.157	—	—
Total	314	—	—	—	—	—	—	—	—	0.741*	0.040*

Abbreviations: *A*, the number of different alleles; *Ar*, the allelic richness; *F*, fixation index; *F*_IS_, inbreeding coefficient; *He*, the expected heterozygosity; *Ho*, the observed heterozygosity; *Ia*, the index of association; *Np*, the number of private alleles; *Ns*, the number of samples; *rD*, the standardized index of association; *uHe*, the unbiased expected heterozygosity.

* *p* < .05.

No significant difference was found in *uHe* per locus at the population or province level. Likewise, no significant correlation was detected between population size (*Ns*) and *Ar* (*r* = .34, adjusted *p* = 1), *Ho* (*r* = .67, adjusted *p* = .23), *He* (*r* = .36, adjusted *p* = 1), *uHe* (*r* = .34, adjusted *p* = 1), and *F* (*r* = −.38, adjusted *p* = 1), respectively. Based on the multilocus LD, and *I*_a_ and *r*_D_, LD was detected among EST‐SSR loci across all populations (*I*_a_ = 0.741, *p* < .05; *r*_D_ = 0.0401, *p* < .05); particularly, in DMS (*I*_a_ = 0.233, *p* < .05; *r*_D_ = 0.016, *p* < .05) and ZJJ (*I*_a_ = 0.336, *p* < .05; *r*_D_ = 0.023, *p* < .05) (Table 4).

### 3.2 Genetic differentiation among populations

High genetic differentiation was found across *P*. *chienii* populations (*F*_ST_ = 0.31; Table 2). Similarly, high genetic differentiation was also detected between populations or provinces, with *F*_ST_ ranging from 0.02 to 0.48 and 0.098 to 0.353 (Tables S5 and S6), respectively.

**TABLE 2:** The partition of EST‐SSR variation of *Pseudotaxus chienii* by analysis of molecular variance (AMOVA) (*p < .05)

Source of variance	*df*	Variance components	Percentage of variation (%)	Phi
Among populations	10	3.416	31.042	(Φ_st_) 0.310*
Among individuals within populations	303	0.705	6.402	(Φ_is_) 0.093*
Within individuals	314	6.884	62.555	(Φ_it_) 0.374*
Total	627	11.005	100	

**FIGURE 2:**
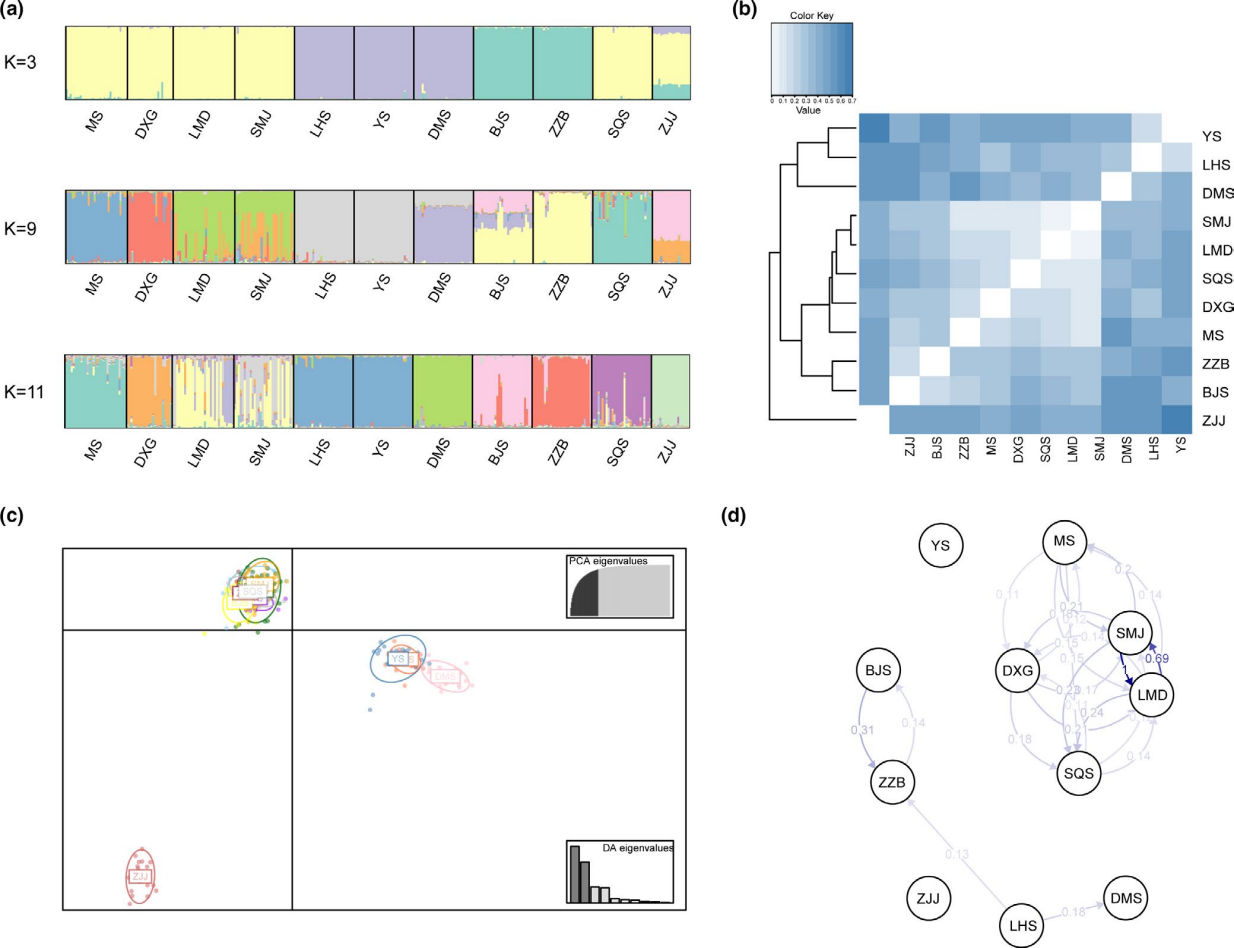
(a) Individual and population memberships to genetic clusters for *K* = 3, 9, and 11 using STRUCTURE. (b) Heatmap of Nei's genetic distance with UPGMA tree between *Pseudotaxus chienii* populations. (c) Clustering results of *Pseudotaxus chienii* populations obtained by discriminant analysis of principal components (DAPC, PCs = 40). (d) The relative migration networks among *Pseudotaxus chienii* populations. Only *Nm* values over 0.1 are shown in the graph

Using STRUCTURE, the optimal clusters were identified as three, nine, and eleven (Figure S1). We selected *K* = 3 as the optimal scenario (Figure 2a). Cluster 1 included populations MS, DXG, LMD, SMJ, SQS, and ZJJ, cluster 2 contained populations LHS, YS, and DMS, and cluster 3 comprised populations BJS and ZZB. When *K* = 9 and 11, only populations LMD and SMJ, or LHS and YS were clustered into the same group as *K* = 3. Populations MS, DXG, LMD, SMJ, and SQS were found highly mixed regardless of *K* value. Certain individuals in population ZJJ were moved from cluster 1 (*K* = 3) into population BJS (*K* = 9) and formed a group themselves (*K* = 11).

All *P*. *chienii* populations were clustered into three groups based on UPGMA (Figure 2b): populations from Guangxi (GX), populations from Zhejiang (ZJ) and Jiangxi (JX), and the single population ZZJ from Hunan (HN). Moreover, Hunan and Guangxi populations were also distinct from the other populations by DAPC (Figure 2c). Forty PCs explained 91.1% of the variance of allelic differences. Based on the membership probability using *K*‐means (Figure S2), populations YS, DMS, and ZJJ were the most distinct with no admixed individuals, while populations MS, DXG, LMD, SMJ, and SQS were highly admixed.

### 3.3 Landscape heterogeneity

Based on PERMANOVA, landscape variables were shown significant difference across the whole distribution of *P*. *chienii* (*p* = .001). Pairwise comparison of variables between provinces also revealed significant difference, such as between Zhejiang (ZJ) and Guangxi (GX), Zhejiang (ZJ) and Jiangxi (JX), Hunan (HN) and Jiangxi (JX), and Hunan (HN) and Guangxi (GX). Similarly, Pairwise comparisons of variables between populations also showed significant difference, except for populations DXG versus LM and BJS versus YS.

### 3.4 Isolation by distance (IBD) and isolation by environment (IBE)

**FIGURE 3:**
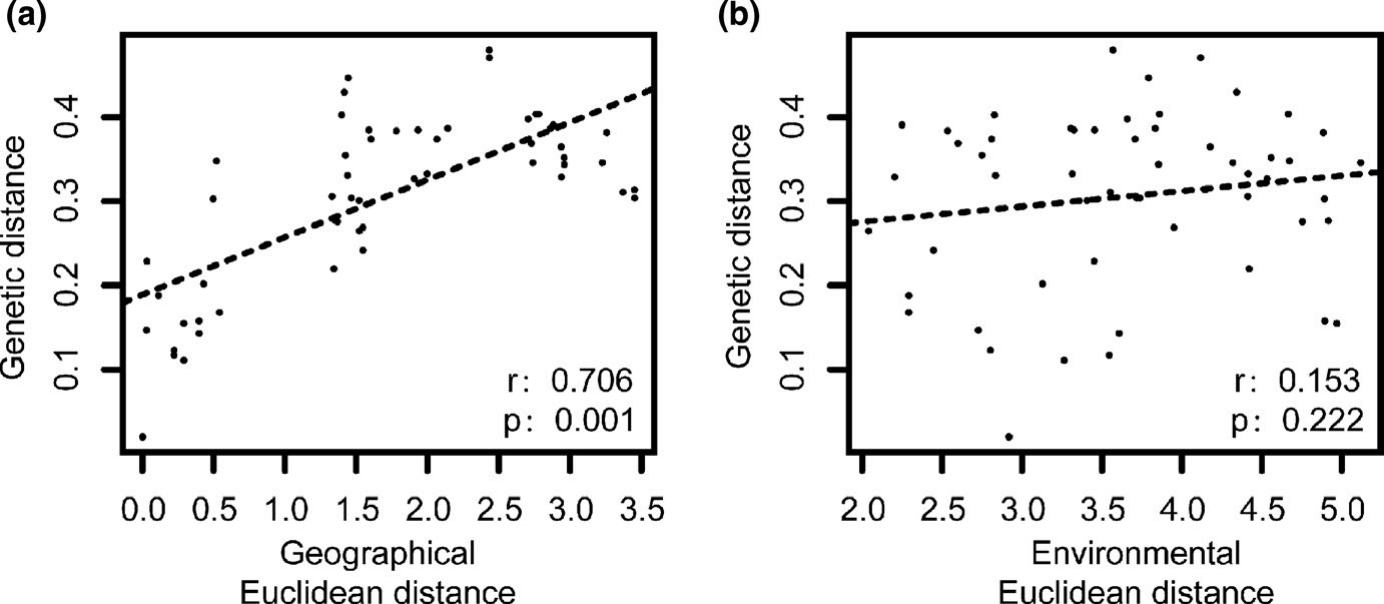
(a) The relationship between genetic distance and geographical distance of *Pseudotaxus chienii*. (b) The relationship between genetic distance and environmental distance of *Pseudotaxus chienii*

Mantel test (Table 3; Figure 3) showed that *P*. *chienii* populations conformed to the pattern of isolation by distance (IBD) but did not to that of isolation by environment (IBE). A significant positive relationship was found between geographical and genetic distance (*r* = .706, *p* < .05), but was not between environmental and genetic distance (*r* = .153, *p* = .222). Partial Mantel tests (Table 3) showed pairwise *F*_ST_ was significantly correlated with geographical distance when controlling for environmental distance (*r* = .698, *p* < .05); but such a significant correlation was not found with environmental distance when controlling for geographical distance (*r* = .055, *p* = .398). As for MMRR analysis (Table 3), when considering geography or environment independently, genetic variation showed a significant correlation with geography (IBD: β_D_ = 0.706, *p* < .05) but not with environment (IBE: β_E_ = 0.153, *p* = .482). When considering geography and environment simultaneously, a similar result was obtained (IBD: β_D_ = 0.699, *p* < .05; IBE: β_E_ = 0.040, *p* = .792).

**TABLE 3:** Results of standard/partial Mantel test and MMRR analysis of *Pseudotaxus chienii*

Test		*R*	*p*	*β*_D_ (*p*)	*β*_E_ (*p*)
Mantel	Gen ~Geo	.706	.**001**		
	Gen ~Env	.153	.222		
Partial Mantel	Gen ~Geo | Env	.698	.**001**		
	Gen ~Env | Geo	.055	.398		
MMRR	Gen ~Geo			0.706 (.**001**)	
	Gen ~Env				0.153 (.482)
	Gen ~Geo + Env			0.699 (.**001**)	0.040 (.792)

Note

Bold font, significant probability.

Abbreivations: Env, environmental distance; Gen, genetic distance; Geo, geographical distance; *β*_D_, the effects of geographical distance on genetic distance; *β*_E_, the effects of environmental distance on genetic distance.

### 3.5 Genetic variation explained by geographical/environmental factors

RDA showed that both geographical and environmental factors had a significant impact on genetic variation, and the environmental factors were more important (Table 4). The combined effects of geographical and environmental factors (i.e., IBD∪IBE) accounted for 38.4% of the total genetic variation, while their intersection (i.e., IBD∩IBE) explained 8.3%. Environmental factors alone contributed more to genetic variation (22.2%) in comparison with geographical factors (8.0%).

**TABLE 4:** Proportion of genetic variation explained by environmental variables (Env, [a]), shared environmental and geographical factors [c], geographical (Geo, [c]), and undetermined component [d]

	Adjusted *R^2^*	*F*	*p*
Env [a]	0.222	17.013	.001
Geo +Env [b]	0.083	—	—
Geo [c]	0.080	20.774	.001
[a + b+c]	0.384	22.691	.001
Residuals [d]	0.616	—	—

### 3.6 Spatial genetic structure

**FIGURE 4:**
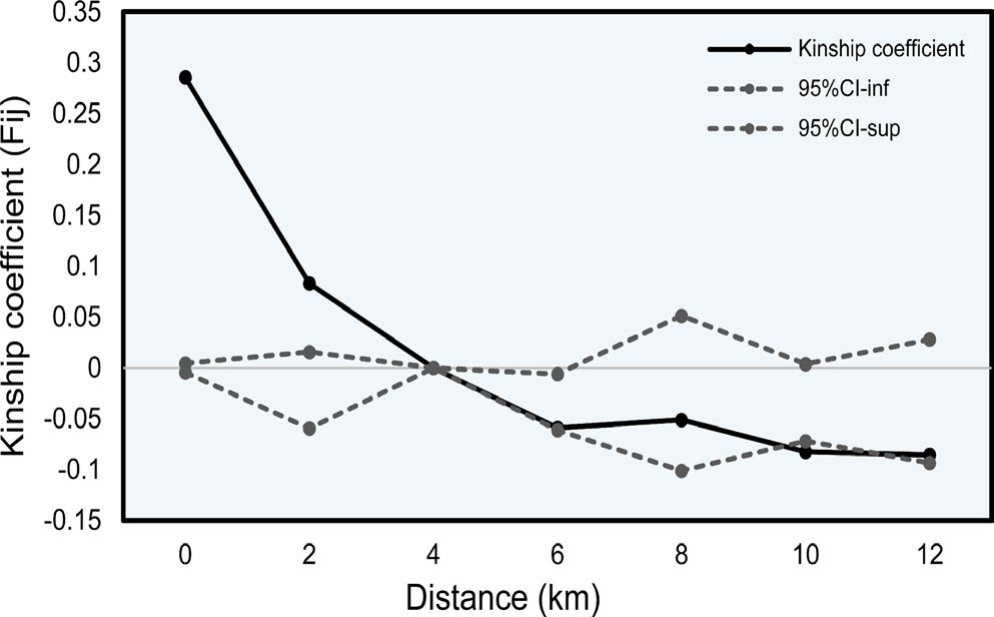
The fine‐scale spatial autocorrelation analysis of *Pseudotaxus chienii*

*Pseudotaxus chienii* populations displayed strong fine‐scale spatial genetic structure within 2 km (*Sp* = 0.048; b_log_ = −0.044, *p* < .05). The *F*_ij_ for pairwise individuals was greater or less than zero when geographical distance was less or greater than 4 km, respectively. A decline tendency of *F*_ij_ indicated that the similarity between individuals became lower with the increase of distance (Figure 4). However, *F*_ij_ was found to be significantly positive only at the intragroup level and within the first distance class (0–2 km; *F*_0_ = 0.286, *p* < .05; *F*_1_ = 0.083, *p* < .05), but became significantly negative within the fifth distance class (8–10 km; *F*_5_ = −0.083, *p* < .05).

Except for having positive values in the first and the fifth distance class, Moran's *I* had negative values in all other distance classes (Figure S3). As shown by the result at the fifth distance class (Moran's *I* = 0.696, *p* = .043), the spatial autocorrelation among *P*. *chienii* populations may extend to 600 km.

### 3.7 Demographic change

The migration networks showed a low level of migration among the 11 *P*. *chienii* populations (Figure 2d; Table S7). Except for populations SMJ and LMD, frequent but relatively low levels of gene flow were detected among populations MS, DXG, LMD, SMJ, and SQS. By contrast, almost negligible gene flow was detected between populations YS and ZJJ and the other populations.

Genetic bottleneck signal was detected in several populations with a significant heterozygosity excess (*p* < .05) (Table S8). It is of note that no population were found to undergo bottleneck under SMM model; by contrast, populations DMS and ZZB were detected to experience a population size reduction under TPM model. Moreover, the lack of bottleneck effect was also indicated by the normal L‐shaped distribution of allele frequency of “mode‐shift” test.

We detected genetic discontinuities in the geographical distribution area of *P*. *chienii*. Three potential spatial boundaries were identified between populations YS and ZZB, YS and ZJJ, and LHS and DMS, respectively (Figure 1).

### 3.8 Candidate selective loci associated with environmental variables

Loci EMS3 and EMS6 were identified as under balance selection and positive selection, respectively (Figure S4). The former was found similar to *AtERF054* (*Arabidopsis thalian* ethylene‐responsive transcription factor ERF054; Evalue = 1.5E‐21), while the latter to *OsCESA7* (cellulose synthase A catalytic subunit 7 of *Oryza sativa* subsp. *japonica*; Evalue = 0) through BLASTN search.

We investigated associations between the two loci and 18 selected landscape variables (Table 5). Eight loci were found to be associated with geographical variables, 13 with soil variables, ten with bioclimatic variables, and eight with ecological variables. The majority of loci were associated with longitude (5), Cu (4), and percent tree cover (PTC, 4). EMS3 alleles of 439 bp and 452 bp were detected significantly associated with six landscape variables: longitude, altitude, PTC, Cu, Bio10 (annual mean temperature of the warmest quarter, and Bio13 (precipitation of the wettest month). EMS6 alleles of 263 bp and 270 bp were detected significantly associated with eight landscape variables: longitude, latitude, PTC, K, Cu, Pb, Bio11 (annual mean temperature of the coldest quarter), and Bio14 (precipitation of the driest month).

**TABLE 5:** Significant associations between candidate‐selected EST‐SSR outliers and landscape variables

Locus	Allele (bp)	SAMβada/LMM																		Total
		Lng	Lat	Alt	K	Na	Fe	Mn	Zn	Cu	Pb	Bio10	Bio11	Bio13	Bio14	PTC	LAI	fPAR	EVI	
EMS3	EMS3_439	*		*			*		*					**			**			6
	EMS3_440											*								1
	EMS3_445																			0
	EMS3_446			*								*								2
	EMS3_451	*																		1
	EMS3_452	**	*					*						**						4
	EMS3_463									***						*				2
	EMS3_464									***						*				2
EMS6	EMS6_263	a	a								a	*	a/**	a	a					7
	EMS6_264																			0
	EMS6_269				a					a/***						a/*				3
	EMS6_270	a	a/*		a/**	a	a			***	a		a/*		a	*		a		11
Total		5	3	2	2	1	2	1	1	4	2	3	2	3	2	4	1	1	0	

Abbreviations: Alt, altitude; EVI, enhanced vegetation index; fPAR, fraction of absorbed photosynthetically active radiation; LAI, leaf area index, enhanced vegetation index; Lat, latitude; Lng, longitude; PTC, percent tree cover.

^a^ Significant correlation of outliers with environmental variables by SAMβada.

* Significant possibility with *p* < .05 by LMM.

** Significant possibility with *p* < .01 by LMM.

*** Significant possibility with *p* < .001 by LMM.

Longitude, Cu, and PTC were the most important variables, associated with the largest number of EMS3 and EMS 6 alleles. Overall, longitude had effects on both loci (although had relatively weak effect on EMS3), while latitude only imposed effects on EMS6. PTC and Cu affected both loci. Altitude had strong effects on EMS3 but relatively weak effects on EMS6. There were four temperature‐ and precipitation‐related variables constituting two combinations having effects on each locus (EMS3: Bio10 and Bio13; EMS6: Bio11 and Bio14), respectively. Moreover, there were more environmental variables associated with EMS6 (e.g., K, Pb, fPAR) than EMS3.

### 3.9 Ecological niche modeling of *P*. *chienii*

An accurate model performance was obtained as indicated by the average AUC 0.967 ± 0.029 for the potential distribution prediction of *P*. *chienii*. Bio14 (the precipitation of driest month) and Bio10 (the mean temperature of warmest quarter) were found to be the key factors in determining the distribution (Table S9), with contribution rates of 64.4% and 20.7%, respectively.

**FIGURE 5:**
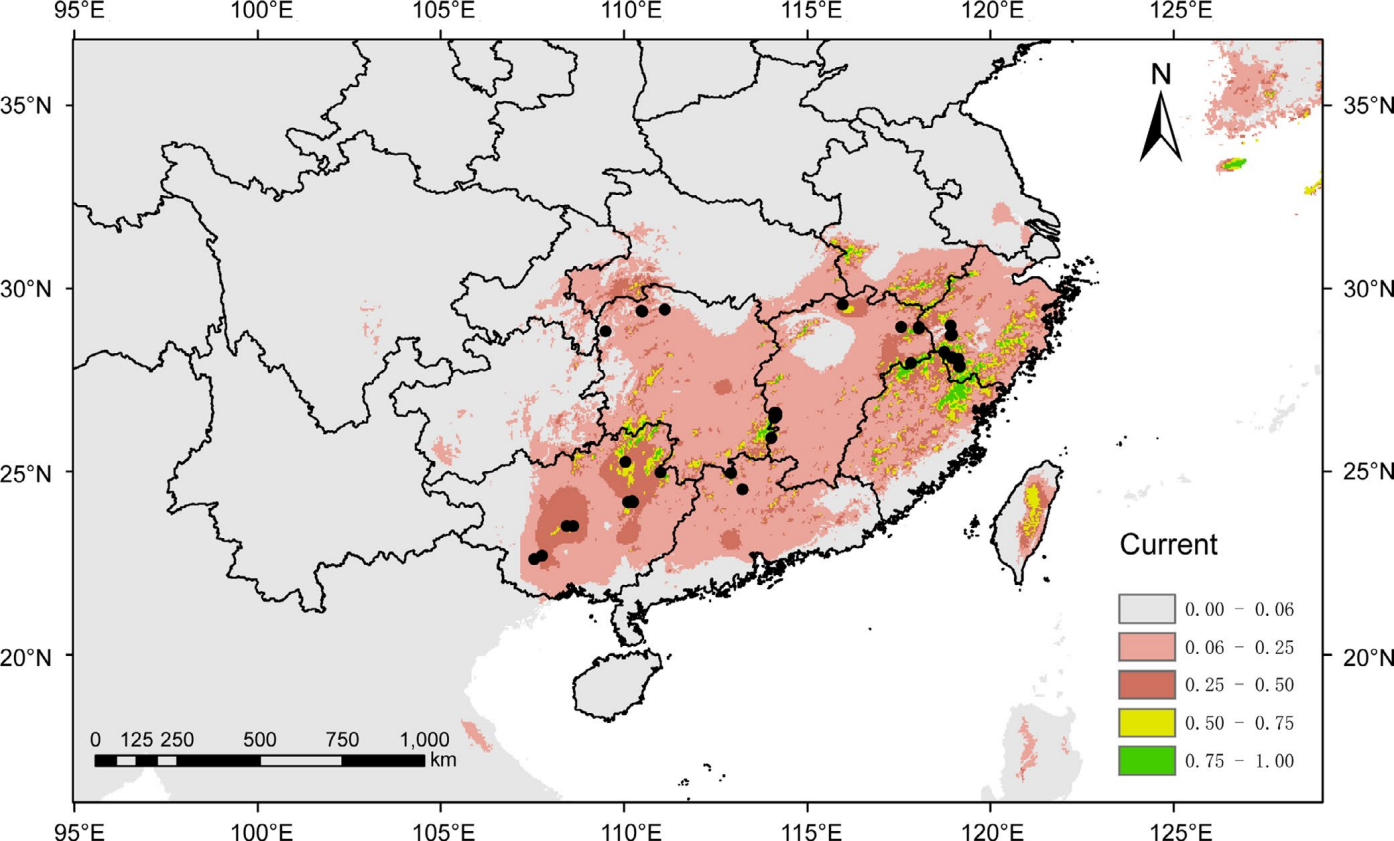
Potential geographical distribution of *Pseudotaxus chienii* in China under current climate condition

**FIGURE 6:**
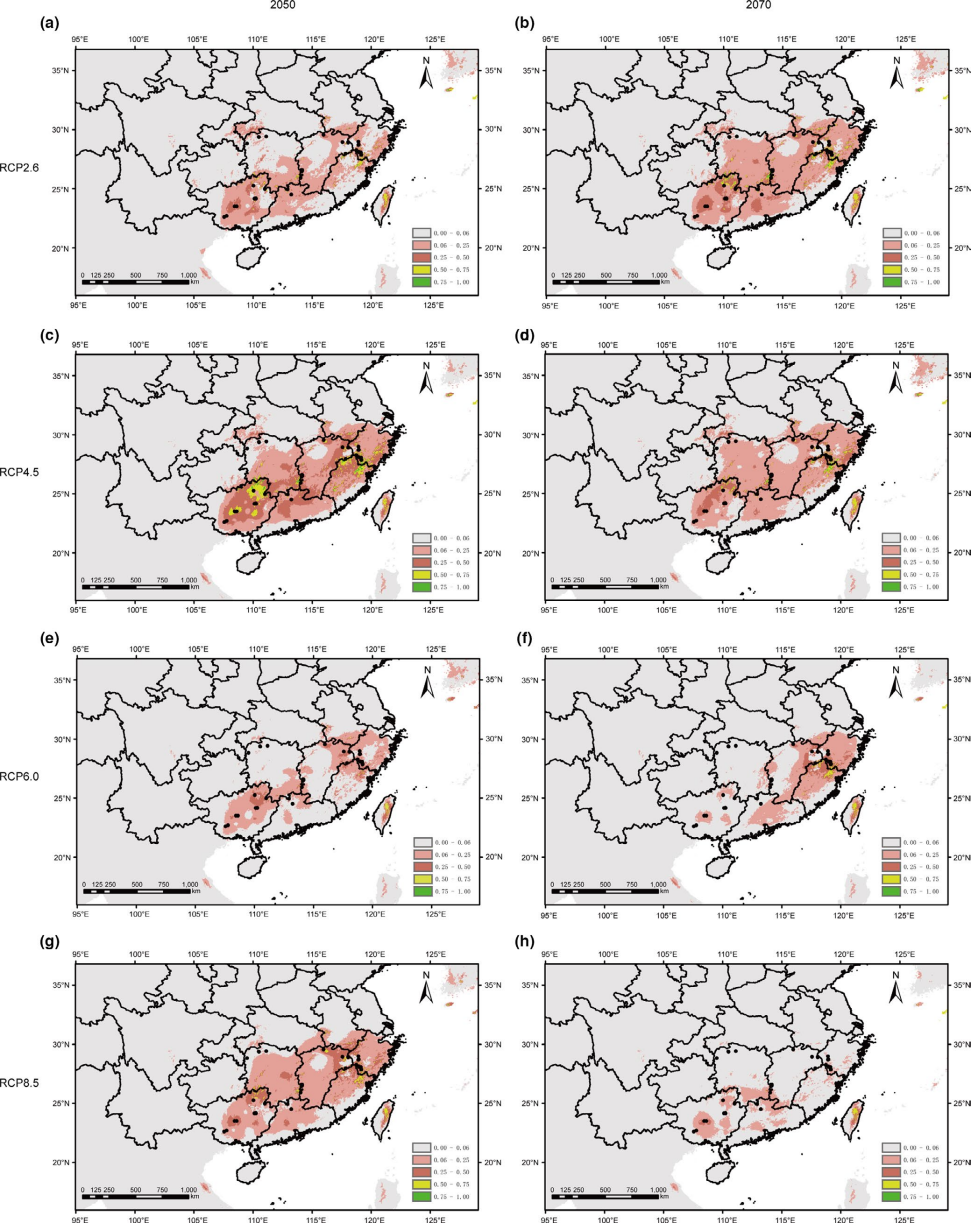
Potential geographical distribution of *Pseudotaxus chienii* in China under future climate condition (a: RCP2.6 to the year 2050; b: RCP2.6 to the year 2070; c: RCP4.5 to the year 2050; d: RCP4.5 to the year 2070; e: RCP6.0 to the year 2050; f: RCP6.0 to the year 2070; g: RCP8.5 to the year 2050; and h: RCP8.5 to the year 2070)

The predicted current suitable area of *P*. *chienii* was consistent with its actual distribution, involving large areas of Guangxi, Guangdong, Hunan, Jiangxi, Fujian, Zhejiang, and Taiwan, and small region of Jiangsu, Anhui, Hubei, Chongqing, Sichuan, and Guizhou (Figure 5). The predicted future distribution showed significant contractions on a small or large scale under different RCP scenarios. Of note, the predicted distribution changes were not consistent in 2050 and 2070 (Figure 6).

## 4 DISCUSSION

This study aims to gain a clearer understanding of how landscape variables affect the local adaptation *P*. *chienii* populations. EST‐SSRs have been applied to investigate the landscape genetics of *P*. *chienii*, a conifer endemic to China. We have examined the population genetic diversity, genetic differentiation, and the spatial genetic structure, performed genome scan to detect outlier loci, conducted selection scan to measure locus‐landscape variable correlations, and dissected the relative effects of landscape factors and demographic history. Moreover, the ecological niche of *P*. *chienii* has been modeled under climate change.

### 4.1 Genetic diversity of *P*. *chienii*

This study has detected a moderate level of EST‐SSR variation (*He* = 0.370) in *P*. *chienii* at the species level. In comparison with other coniferous species (Table S10), its average expected heterozygosity is lower than *Torreya grandis* (*He* = 0.432) (Zeng et al., 2018), *Pinus massoniana* (*He* = 0.5717) (Zhang et al., 2014), *Picea abies* (*He* = 0.616) (Stojnić et al., 2019), and *P*. *likiangensis* (*He* = 0.7186) (Cheng et al., 2014); close to *P*. *dabeshanensis* (*He* = 0.36) (Zhang et al., 2016) and *Amentotaxus argotaenia* (*He* = 0.39) (Ruan et al., 2019); and higher than *A*. *formosana* (*He* = 0.1993) (Li et al., 2016), *P*. *bungeana* (*He* = 0.205) (Duan et al., 2017), and *A*. *yunnanensis* (*He* = 0.3343) (Li et al., 2016). It has been suggested that levels of genetic variation in conifers are influenced by a variety of factors including lifespan, reproductive system, seed dispersal mechanisms, geographical distribution range, life forms, demographic history, natural selection, and mutation rate (Hamrick et al., 1992; Su et al., 2009; Wang et al., 2020).

Our analysis indicates that *P*. *chienii* populations enable to maintain moderate level of EST‐SSR variation although they are impacted by inbreeding. Nine of 11 (81.82%) of the populations have the estimated inbreeding levels ranging from 0.009 to 0.199 (Table 1). These results are not unexpected considering the current *P*. *chienii* populations are usually of small size (several to tens) and geographically scattered and isolated (Su et al., 2009). Importantly, because *Pseudotaxus chienii* plants tend to grow in the understory of forests (Fu & Jin, 1992), this may hinder long‐distance pollen dispersal and enhance inbreeding as well. Similar results have been observed in other related yews like *T*. *baccata* (Chybicki et al., 2011), *Taxus wallichiana* var. *mairei* (Zhang & Zhou, 2013), and *T*. *yunnanensis* (Miao et al., 2016). In addition, a significant fine‐scale spatial genetic structure was detected within 2 km (Figure 4), which also implies gene flow occurring between individuals from adjacent populations. Our results suggest that *P*. *chienii*, as an “old rare species” which has long been naturally fragmented (Fu et al., 1999; Hilfiker et al., 2004; Su et al., 2009), appears to have the potential to maintain its functional genetic variation. In this respect, the detection of outlier locus EM3 underlies the implication of balancing selection to preserve EST‐SSR variation (Figure S4).

As a tertiary relict species, the distribution of *P*. *chienii* may have been seriously affected by the Quaternary glacial–interglacial climate changes (Xu et al., 2008). In the meanwhile, its populations may have undergone both expansion and shrinkage (Table S8) (Zhang et al., 2020). Hence, it cannot be excluded that population demographic history is relative to the EST‐SSR variation.

### 4.2 High levels of population genetic differentiation of *P*. *chienii*

*P. chienii* populations exhibit a high level of genetic differentiation across the distribution range (*F*_ST_ = 0.31; Table 2), in comparison with results observed in other conifers like *P*. *resinosa* (*F*_ST_ = 0.280) (Boys et al., 2005), *T*. *chinensis* (*F*_ST_ = 0.189), and *T*. *wallichiana* (*F*_ST_ = 0.156) (Vu et al., 2017). The high among‐population genetic differentiation of *P*. *chienii* populations has also been revealed by using RAPD (Wang et al., 2006) and ISSR markers (Su et al., 2009). Possible factors contributing this high genetic differentiation may include a low level of migration among populations (Figure 2d; Table S7); spatial barriers (Figure 1); bottlenecks (Table S8); small population size (Fu & Jin, 1992); a long evolutionary history, and genetic drift (Su et al., 2009); limited pollination (Fu & Jin, 1992); a wide and disjunct distribution (Fu & Jin, 1992; Su et al., 2009); and IBD (Table 3; Figure 3).

It is of note that a relatively weak genetic differentiation was detected between populations LMD and SMJ (*F*_ST_ = 0.02; Table S5). FSGS analysis reveals a significant spatial genetic structure within 2 km in *P*. *chienii* populations. More importantly, their FSGS intensity (*Sp* = 0.0483) was much higher than that of other conifers including *T*. *baccata* (*Sp* = 0.006, 0.009) (Chybicki et al., 2011), *T*. *yunnanensis* (*Sp* = 0.001) (Miao et al., 2016), and *P*. *omorika* (*Sp* = 0.009) (Aleksić et al., 2017). Given that the geographical distance LMD and SMJ is less than 2 km, one possible explanation for their weak between‐population genetic differentiation is that a short‐distance dispersal of pollen or seed remains to be effective for *P*. *chienii*.

### 4.3 Important landscape variables potentially driving the adaptive genetic differentiation of *P*. *chienii*

Two (10%) EST‐SSR loci EMS3 and EMS6 were simultaneously identified as candidate outliers by running BAYESCAN and FDIST; and they have also been detected significantly associated with landscape variables. The consistent identification by three different methods strongly supports that EMS3 and EMS are loci potentially under selection. EMS3 shows sequence similarity to *AtERF054* (*Arabidopsis thalian* ethylene‐responsive transcription factor ERF054) and EM6 to *OsCESA7* (cellulose synthase A catalytic subunit 7 of *Oryza sativa* subsp. *japonica*). Notably, EMS6 is inferred to be positively selected, whose alleles are significantly associated with (1) eight individual variables: longitude, latitude, PTC, K, Cu, Pb, Bio11 (annual mean temperature of the coldest quarter), and Bio14 (precipitation of the driest month), and (2) the combined effects of Bio11 and Bio14.

Our results highlight the potential of specific soil metal content as the driving factor of local adaptation for *P*. *chienii* populations. First, Cu was found to be crucial. Cu is an essential nutrient element, functioning as a cofactor in more than 100 metalloproteins (Yruela, 2009) and participating in many physiological processes including photosynthesis, respiration, carbon and nitrogen metabolism, protection against oxidative stress, hormone signaling, and cell wall metabolism (DalCorso et al., 2014). Cu deficiency may reduce the rates of photosynthesis and carbohydrate synthesis, whereas excess suppresses the root absorption of Mn and Fe (Ivanov et al., 2016). Second, plants require K in relatively large amounts. K has a high mobility in plant cells and in long‐distance transport through the xylem and phloem (Meena et al., 2016). It is essential for plant growth and metabolisms, functioning in the control of water status, promotion of water absorption, maintenance of osmotic tension and turgor, and regulation of the activity of stomata cells. K also has a critical role in photosynthesis, in the production and translocation of carbohydrate, and in stress responses (El Sayed et al., 2019; Wang et al., 2013). Third, Pb represents a harmful nonessential element, posing serious threats to plant growth (Patra et al., 2004). In these contexts, it is reasonable to postulate that selective pressures from soil metal contents may contribute to the genetic structuring of *P*. *chienii* populations.

The adaptive genetic differentiation of EMS6 is also associated with longitude, latitude, PTC, Bio11 (annual mean temperature of the coldest quarter), Bio14 (precipitation of the driest month), and the combined effects of Bio11 and Bio14. Particularly, Bio14 has been identified as the factor contributing the most (64.4%) in determining the distribution of *P*. *chienii*. These results are consistent with the ecological characteristics of *P*. *chienii* (i.e., preferentially growing under dense canopies in montane forests and mainly occurring in humid habitats) (Fu & Jin, 1992). Nevertheless, of longitude and latitude it cannot be excluded that the association is caused by the collinearity with other landscape variables.

Ecological niche modeling of *P*. *chienii* predicts that *P*. *chienii* may experience significant range contractions under future climate change scenarios (Figure 6). This information, in junction with the identified landscape variables potentially driving the adaptation, provides useful data to develop a conservation action plan for *P*. *chienii*.

## 5 CONCLUSION

This study firstly integrated EST‐SSRs and landscape genetics analyses to investigate the population genetic pattern of *P*. *chienii*. *P*. *chienii* was found to maintain a moderate level of genetic variation and a high level of genetic differentiation. Its populations showed a IBD pattern and a strong fine‐scale spatial genetic structure within 2 km. A putatively adaptive locus EMS6 was identified, functionally annotated, and found to present significant associations with soil Cu, K, and Pb content and the combined effects of temperature and precipitation. In addition, *P*. *chienii* was predicted to experience significant range reductions in future climate change scenarios. These results lend support to the implication of landscape variables in the adaptive genetic differentiation in *P*. *chienii*. They would also be useful for developing a conservation action plan for the plant.

## CONFLICT OF INTEREST

The authors declare no conflicts of interest.

## AUTHOR CONTRIBUTIONS

**Shufeng Li:** Formal analysis (equal); Writing‐original draft (equal). **Zhen Wang:** Investigation (equal); Writing‐review & editing (equal). **Yingjuan Su:** Project administration (lead); Writing‐review & editing (supporting). **Ting Wang:** Project administration (equal); Writing‐review & editing (equal).

## Supporting information

Supplementary Material

Figure S1

Figure S2

Figure S3

Figure S4

## Data Availability

The datasets used for this study are available through Dryad at the time of publication (https://doi.org/10.5061/dryad.95x69p8kp).
