# Supplementary material for: EST‐SSR‐based landscape genetics of Pseudotaxus chienii, a tertiary relict conifer endemic to China
Source: Ecol Evol. 2021 Jun 15;11(14):9498–515. doi: 10.1002/ece3.7769 (PMC8293779; doi:10.1002/ece3.7769)
Supplement: Supplementary file 1 — Supplementary Material [file ECE3-11-9498-s001.docx]

Supplementary Material

Appendix 1:

TABLE S1 The geographical information of 11 *Pseudotaxus chienii* populations

| Provenance and code | Population location and code | Geographical coordinate | Altitude (m) |
| --- | --- | --- | --- |
| Zhejiang (ZJ) | Maoshan (MS) | 118°58'23''E, 28°06'08''N | 1120 |
|  | Daxiagu (DXG) | 119°10'24''E, 27°52'49''N | 1500 |
|  | Longmending (LMD) | 118°57'13''E, 28°43'38''N | 1200 |
|  | Shuimenjian (SMJ) | 118°57'32''E, 28°43'42''N | 830 |
| Guangxi (GX) | Lianhuashan (LHS) | 110°06'47''E, 24°09'28''N | 1026 |
|  | Yinshan Park (YS) | 110°14'53''E, 24°09'15''N | 1050 |
|  | Damingshan (DMS) | 108°26'12''E, 23°29'54''N | 1240 |
| Jiangxi (JX) | Bijianshan (BJS) | 114°09'41''E, 26°30'35''N | 1340 |
|  | Zizhuba (ZZB) | 114°06'22''E, 26°27'18''N | 1300 |
|  | Sanqingshan (SQS) | 118°04'09''E, 28°54'19''N | 1500 |
| Hunan (HN) | Zhangjiajie (ZJJ) | 110°28'56''E, 29°23'12''N | 1055 |

TABLE S2 The information of 20 pairs of EST-SSR primers

| Locus | Repeat motif | Forward primers (5’-3’) | Reverse primers (5’-3’) | Position | *Ta* (°C) |
| --- | --- | --- | --- | --- | --- |
| EMS1 | (ATA)5 | GAGGGATACAGAAGCACAG | TATGACAAACCCAAACGAG | CDS | 56 |
| EMS2 | (ATA)6 | GACAACGGCAAAGGAGGAAT | GCGATAGCCACCAAAGACAT | CDS | 58 |
| EMS3 | (CTCCTG)5 | TGCGGTTCAGTAACAGTCCTTC | TCCCCCACCTCTTCCCAG | CDS | 58 |
| EMS4 | (ATGCAG)7 | TGTGTGAAAGGACAAGGCGT | GCACCCTATTCACCCGAGAT | UTR5 | 56 |
| EMS5 | (CTG)5 | CCCCTCATTGACAGGTTC | AAGATAGTCGGGACACCAAG | CDS | 56 |
| EMS6 | (AAGG)5 | CACGCCCACCATAGTTGT | GGAGGAAGATGTCGTTGAAG | UTR3 | 58 |
| EMS7 | (TCC)5 | TAAGTGGCTGCTGCATCACA | TACAGCAGCAGCAGAGCTTT | UTR5 | 58 |
| EMS8 | (CCT)11 | GACCTCTTACCAGCTGCGAG | ACCACCGGTTTCAGTTTCGT | UTR5 | 62 |
| EMS9 | (ACC)6 | TGTGCCAGTACTGCTACTGC | TGAATGCGTGCGGAAACAAG | CDS | 57 |
| EMS10 | (GGA)8 | GATGCCGCTGGTTTCAATCC | GCCGTACCGATTGGGATCAT | CDS | 57 |
| EMS11 | (CTC)8 | GAGTGGGAGACGAAGAGTGC | CGAAGTGGGCTGCAACAATG | UTR3 | 57 |
| EMS12 | (GAA)5 | AGCTGCAAGGCTACACAGAG | CAATCCCGGGCCTGTTAGAA | CDS | 57 |
| EMS13 | (CCACCG)6 | ACCTATCACCTCCTCGACCC | CCGTTCCATCACTGTGGACA | CDS | 55 |
| EMS14 | (TCC)8 | GGGCCATCCTCTTCCTCAAC | CTCGACACTGCTCCACATCT | UTR5 | 57 |
| EMS15 | (AGG)5 | GGTCGAGTACGTGGTGGTTT | GCCTGCGCTGTCATAAACTG | CDS | 57 |
| EMS16 | (CAGAAG)5 | CGCTCCAACGAATCCAACC | TAATGCCATCCGCACAACC | CDS | 57 |
| EMS17 | (GGCACC)5 | GAATTTGAAGCACGGCCTCA | GAGTGCCCTGCTTTCTGGAT | CDS | 57 |
| EMS18 | (CTT)6 | ACGCCACGTTAGGACACAAT | CCTAGATCAAGAGCGGCCTG | UTR5 | 57 |
| EMS19 | (CGG)7 | CTGTCAACAAGCGGCTTTCC | AGAGCCGGGGGAAAATTGAG | CDS | 57 |
| EMS20 | (GGC)7 | CCCATCTGAACCCACGCTAA | AAAGCGCTCATGCCCAAAAC | UTR5 | 57 |

*Ta*, annealing temperature.

TABLE S3 The frequency of null alleles for each marker in *Pseudotaxus chienii* populations

| Pop | Locus | *r* |
| --- | --- | --- |
| MS | EMS5 | 0.230 |
|  | EMS6 | 0.226 |
|  | EMS8 | 0.153 |
|  | EMS17 | 0.337 |
| DXG | EMS5 | 0.386 |
|  | EMS11 | 0.250 |
|  | EMS17 | 0.397 |
| LMD | EMS5 | 0.293 |
|  | EMS7 | 0.268 |
|  | EMS8 | 0.115 |
|  | EMS14 | 0.270 |
|  | EMS17 | 0.356 |
|  | EMS19 | 0.255 |
| SMJ | EMS5 | 0.411 |
|  | EMS8 | 0.220 |
|  | EMS17 | 0.377 |
|  | EMS19 | 0.229 |
| LHS | EMS5 | 0.325 |
|  | EMS15 | 0.224 |
|  | EMS17 | 0.183 |
| YS | EMS2 | 0.224 |
|  | EMS12 | 0.262 |
|  | EMS15 | 0.166 |
|  | EMS17 | 0.156 |
| DMS | EMS5 | 0.224 |
|  | EMS6 | 0.342 |
|  | EMS15 | 0.414 |
|  | EMS17 | 0.189 |
|  | EMS19 | 0.273 |
| BJS | EMS2 | 0.166 |
|  | EMS5 | 0.208 |
|  | EMS6 | 0.265 |
|  | EMS8 | 0.188 |
|  | EMS10 | 0.166 |
|  | EMS12 | 0.214 |
|  | EMS14 | 0.296 |
|  | EMS15 | 0.166 |
|  | EMS17 | 0.208 |
| ZZB | EMS5 | 0.407 |
|  | EMS8 | 0.183 |
|  | EMS9 | 0.166 |
|  | EMS10 | 0.166 |
|  | EMS13 | 0.095 |
|  | EMS15 | 0.384 |
|  | EMS17 | 0.394 |
|  | EMS19 | 0.151 |
| SQS | EMS5 | 0.279 |
|  | EMS14 | 0.394 |
|  | EMS15 | 0.181 |
|  | EMS17 | 0.342 |
|  | EMS19 | 0.275 |
| ZJJ | EMS5 | 0.323 |
|  | EMS6 | 0.288 |
|  | EMS10 | 0.315 |
|  | EMS13 | 0.387 |
|  | EMS15 | 0.270 |
|  | EMS17 | 0.353 |

TABLE S4 The result of Hardy-Weinberg test for *Pseudotaxus chienii* populations (ns = not significant, * *P* < 0.05, ** *P* < 0.01, *** *P* < 0.001)

| Locus | MS | DXG | LMD | SMJ | LHS | YS | DMS | BJS | ZZB | SQS | ZJJ | Total |
| --- | --- | --- | --- | --- | --- | --- | --- | --- | --- | --- | --- | --- |
| EMS1 | ns | ns | *** | *** | - | *** | - | *** | * | ns | - | *** |
| EMS2 | - | - | ns | ns | - | *** | - | * | - | - | - | *** |
| EMS3 | ns | * | *** | *** | ** | ns | * | ** | *** | *** | ns | *** |
| EMS4 | - | - | ns | ns | ns | ns | ns | ns | ns | ns | - | 0.9821 |
| EMS5 | *** | *** | *** | *** | *** | - | *** | *** | *** | *** | *** | *** |
| EMS6 | *** | ns | ns | ns | - | - | *** | *** | - | ns | *** | *** |
| EMS7 | ns | ns | *** | ns | ns | * | ns | ns | ns | ns | ns | * |
| EMS8 | ** | * | ** | *** | - | ns | ns | *** | *** | ns | - | *** |
| EMS9 | *** | ** | ** | ** | - | ns | ** | - | * | ns | - | *** |
| EMS10 | ns | ns | ns | ns | - | ns | - | * | * | ns | *** | ** |
| EMS11 | ns | ** | ns | * | ns | ns | ns | ns | * | ns | ns | * |
| EMS12 | - | ns | ns | ns | ns | *** | *** | *** | ns | ns | ns | *** |
| EMS13 | ns | ns | * | * | * | ns | ns | ns | ** | ** | *** | *** |
| EMS14 | - | - | *** | ns | - | - | - | *** | - | *** | ns | *** |
| EMS15 | ns | *** | ** | *** | *** | * | *** | * | *** | *** | ** | *** |
| EMS16 | * | ns | ns | ns | ns | ns | * | * | ns | * | * | ** |
| EMS17 | *** | *** | *** | *** | *** | *** | *** | ** | *** | *** | *** | *** |
| EMS18 | ns | ns | - | - | ns | ns | ns | ** | ns | - | ns | * |
| EMS19 | ns | ns | *** | *** | ns | *** | *** | ns | ns | *** | ** | *** |
| EMS20 | ** | ns | ns | ns | *** | *** | *** | *** | *** | *** | * | *** |

TABLE S5 Geographical distance (km, upper diagonal) and genetic differentiation estimates (*F_st_*, lower diagonal) between *Pseudotaxus chienii* populations

| Pop | MS | DXG | LMD | SMJ | LHS | YS | DMS | BJS | ZZB | SQS | ZJJ |
| --- | --- | --- | --- | --- | --- | --- | --- | --- | --- | --- | --- |
| MS |  | 31.6 | 69.6 | 69.7 | 988.0 | 976.1 | 1173.1 | 507.8 | 515.2 | 125.7 | 840.8 |
| DXG | 0.188 |  | 96.7 | 96.7 | 996.2 | 984.1 | 1181.7 | 519.1 | 526.3 | 157.2 | 865.3 |
| LMD | 0.155 | 0.158 |  | 0.5 | 1017.0 | 1005.5 | 1200.8 | 533.2 | 541.0 | 88.5 | 827.4 |
| SMJ | 0.111 | 0.143 | 0.020 |  | 1017.5 | 1006.1 | 1201.4 | 533.7 | 541.5 | 89.0 | 827.9 |
| LHS | 0.404 | 0.369 | 0.352 | 0.344 |  | 13.7 | 185.8 | 484.2 | 476.3 | 952.0 | 583.2 |
| YS | 0.346 | 0.398 | 0.365 | 0.329 | 0.229 |  | 198.4 | 473.0 | 465.1 | 941.1 | 582.9 |
| DMS | 0.382 | 0.346 | 0.314 | 0.304 | 0.303 | 0.348 |  | 667.7 | 659.9 | 1134.2 | 686.4 |
| BJS | 0.220 | 0.306 | 0.301 | 0.265 | 0.447 | 0.355 | 0.385 |  | 8.2 | 468.4 | 483.1 |
| ZZB | 0.276 | 0.277 | 0.269 | 0.242 | 0.430 | 0.403 | 0.327 | 0.147 |  | 476.4 | 483.2 |
| SQS | 0.202 | 0.168 | 0.117 | 0.123 | 0.391 | 0.387 | 0.311 | 0.331 | 0.304 |  | 739.5 |
| ZJJ | 0.374 | 0.387 | 0.333 | 0.333 | 0.480 | 0.471 | 0.404 | 0.385 | 0.374 | 0.384 |  |

TABLE S6 Pairwise genetic differentiation (*F_st_* ) values between four provinces*.*

| Provenance | ZJ | GX | JX | HN |
| --- | --- | --- | --- | --- |
| ZJ |  | 0.229 | 0.098 | 0.303 |
| GX | 0.229 |  | 0.217 | 0.353 |
| JX | 0.098 | 0.217 |  | 0.279 |
| HN | 0.303 | 0.353 | 0.279 |  |

TABLE S7 *Nm* estimates between *Pseudotaxus chienii* populations (direction: left to right), for example, *Nm* for MS to DXG was 0.113 and *Nm* for DXG to MS was 0.122.

|  | MS | DXG | LMD | SMJ | LHS | YS | DMS | BJS | ZZB | SQS | ZJJ |
| --- | --- | --- | --- | --- | --- | --- | --- | --- | --- | --- | --- |
| MS |  | 0.113 | 0.152 | 0.207 | 0.022 | 0.031 | 0.030 | 0.079 | 0.066 | 0.151 | 0.040 |
| DXG | 0.122 |  | 0.208 | 0.173 | 0.025 | 0.018 | 0.025 | 0.039 | 0.048 | 0.180 | 0.048 |
| LMD | 0.142 | 0.137 |  | 0.688 | 0.032 | 0.024 | 0.042 | 0.054 | 0.076 | 0.237 | 0.045 |
| SMJ | 0.196 | 0.177 | 1.000 |  | 0.028 | 0.020 | 0.039 | 0.062 | 0.070 | 0.232 | 0.044 |
| LHS | 0.063 | 0.057 | 0.063 | 0.039 |  | 0.073 | 0.178 | 0.061 | 0.129 | 0.050 | 0.055 |
| YS | 0.058 | 0.029 | 0.056 | 0.029 | 0.093 |  | 0.049 | 0.036 | 0.033 | 0.054 | 0.032 |
| DMS | 0.028 | 0.034 | 0.063 | 0.045 | 0.045 | 0.024 |  | 0.020 | 0.051 | 0.042 | 0.031 |
| BJS | 0.070 | 0.040 | 0.051 | 0.056 | 0.033 | 0.064 | 0.031 |  | 0.311 | 0.053 | 0.046 |
| ZZB | 0.037 | 0.038 | 0.051 | 0.063 | 0.032 | 0.034 | 0.059 | 0.143 |  | 0.041 | 0.036 |
| SQS | 0.079 | 0.114 | 0.141 | 0.116 | 0.023 | 0.023 | 0.026 | 0.032 | 0.040 |  | 0.041 |
| ZJJ | 0.059 | 0.062 | 0.078 | 0.080 | 0.018 | 0.021 | 0.030 | 0.053 | 0.051 | 0.078 |  |

TABLE S8 Results of the bottleneck test for 11 *Pseudotaxus chienii* populations

| Pop | *p*-value | | | Mode shift |
| --- | --- | --- | --- | --- |
|  | TPM |  | SMM |  |
| MS | 0.517 |  | 0.935 | normal L-shaped |
| DXG | 0.694 |  | 0.913 | normal L-shaped |
| LMD | 0.929 |  | 1.000 | normal L-shaped |
| SMJ | 0.414 |  | 0.871 | normal L-shaped |
| LHS | 0.826 |  | 0.967 | normal L-shaped |
| YS | 0.798 |  | 0.975 | normal L-shaped |
| DMS | **0.015** |  | 0.316 | normal L-shaped |
| BJS | 0.877 |  | 0.995 | normal L-shaped |
| ZZB | **0.020** |  | 0.290 | normal L-shaped |
| SQS | 0.414 |  | 0.871 | normal L-shaped |
| ZJJ | 0.726 |  | 0.946 | normal L-shaped |

TPM, two-phase mutation model; SMM, stepwise mutation model; *p*-value, probability of one-tailed Wilcoxon signed-rank test of heterozygote excess; bold font, significant probability.

TABLE S9 Estimates of relative contributions of the environmental variables to the Maxent model

| Bioclimate variables | Percent contribution |
| --- | --- |
| Precipitation of driest month (Bio14) | 64.4 |
| Mean temperature of warmest quarter (Bio10) | 20.7 |
| Precipitation of warmest quarter (Bio18) | 9.3 |
| Temperature annual range (Bio5-Bio6) (Bio7) | 4.7 |
| Mean diurnal range (mean of monthly (max temp - min temp)) (Bio2) | 0.8 |

Table S10 Comparison of genetic diversity among several gymnosperms (estimated by EST-SSR markers).

| Species | Family | N | Ho | He | IUCN | Endemic | Reference |
| --- | --- | --- | --- | --- | --- | --- | --- |
| *Pseudotaxus chienii* | Taxaceae | 20 | 0.341 | 0.37 | VU | China | The study |
| *Amentotaxus argotaenia* | Taxaceae | 22 | 0.25 | 0.39 | NT |  | Ruan et al., 2019 |
| *Ametotaxus argotaenia* | Taxaceae | 18 | 0.297 | 0.394 | NT |  | Li et al., 2016 |
| *Ametotaxus formosana* | Taxaceae | 18 | 0.053 | 0.1993 | EN | China |  |
| *Ametotaxus yunnanensis* | Taxaceae | 18 | 0.2683 | 0.3343 | VU |  |  |
| *Ametotaxus poilanei* | Taxaceae | 18 | 0.359 | 0.347 | VU |  |  |
| *Torreya grandis* | Taxaceae | 11 | 0.54 | 0.432 |  | China | Zeng et al., 2018 |
| *Picea abies* | Pinaceae | 8 | - | 0.616 |  |  | Stojnić et al., 2019 |
| *Pinus dabeshanensis* | Pinaceae | 10 | 0.46 | 0.36 | EN | China | Zhang et al., 2016 |
| *Pinus dabeshanensis* | Pinaceae | 13 | 0.481 | 0.458 | EN | China | Xiang et al., 2015 |
| *Pinus bungeana* | Pinaceae | 19 | 0.206 | 0.205 | EN | China | Duan et al., 2017 |
| *Pinus massoniana* | Pinaceae | 14 | 0.3373 | 0.5717 |  |  | Zhang et al., 2014 |
| *Pinus hwangshanensis* | Pinaceae | 14 | 0.3363 | 0.4887 |  |  |  |
| *Picea likiangensis* | Pinaceae | 6 | 0.5798 | 0.7186 |  |  | Cheng et al., 2014 |
| *Pinus ponderosa* | Pinaceae | 6 | 0.3617 | 0.6383 |  |  | Lesser et al., 2012 |
| *Picea omorika* | Pinaceae | 5 | 0.65 | 0.603 |  |  | Aleksić et al., 2017 |

VU, Vulnerable; NT, Near Threatened; EN, Endangered.

Lesser, M. R., Parchman, T. L., & Buerkle, C. A. (2012). Cross-species transferability of SSR loci developed from transciptome sequencing in lodgepole pine. *Molecular Ecology Resources*, *12*, 448–455.

**Appendix 2:**

**Landscape variables**

The six ecological variables were: elevation, percent tree cover (PTC) from moderate resolution imaging spectroradiometer (MODIS) dataset MOD44B (250 m resolution; 2000-2011), normalized difference vegetation index (NDVI) and enhanced vegetation index (EVI) from MOD13A2 (500 km resolution; 2001-2011), and leaf area index (LAI) and fraction of absorbed photosynthetically active radiation (fPAR) from MOD15A2 dataset (500 m resolution; 2001-2011). All the MODIS datasets were acquired from NASA’s Land Processes Distributed Active Archive Center (LP DAAC, http://lpdaac.usgs.gov). We adopted maximum value composites in ENVI 5.3 software to acquire the annual layers of PTC, NDVI, EVI, LAI and fPAR. In addition, 20 soil variables were from our Plant Evolutionary Genetics Lab (Wang et al., 2006). Nineteen bioclimate variables (1950-2000) were downloaded from the WorldClim version 1.4 (http://www.worldclim.org/) at 2.5 arc-minutes resolution (Hijmans et al., 2005). ArcGIS 10.2 was used to extract the five ecological (PTC, NDVI, EVI, LAI, and fPAR) and 19 bioclimate variables. Elevation was regarded as a climatic variable in this study due to representing vertical variation of microclimate in landscape (Wanderley et al., 2018).

Wang, T., Su, Y. J., OuYang, P. Y., Huang, H. W., Chen, C. Q., Zeng, X. M., … Hu, S. Q. (2006). Using RAPD markers to detect the population genetic structure of *Pseudotaxus chienii* (Taxaceae), an endangered and endemic conifer in China. *Acta Ecologica Sinica*, *26*, 2313–2321.

Wanderley, A. M., Machado, I. C. S., de Almeida, E. M., Felix, L. P., Galetto, L., Benko-Iseppon, A. M., & Sork, V. L. (2018). The roles of geography and environment in divergence within and between two closely related plant species inhabiting an island-like habitat. *Journal of Biogeography*, *45*, 381–393.

**Appendix 3:**

FIGURE LEGENDS of Figures S1-S4

Figure S1: Plot of the value of *ΔK* for each *K* from STRUCTURE analysis.

Figure S2: Clustering results of *Pseudotaxus chienii* populations analyzed by *k*-means method.

Figure S3: The large-scale spatial autocorrelation analysis of *Pseudotaxus chienii*.

Figure S4: Outliers detected by BAYESCAN (A) and FDIST (B).
