# Supplementary figures and images for: EST‐SSR‐based landscape genetics of Pseudotaxus chienii, a tertiary relict conifer endemic to China
Source: Ecol Evol. 2021 Jun 15;11(14):9498–515. doi: 10.1002/ece3.7769 (PMC8293779; doi:10.1002/ece3.7769)

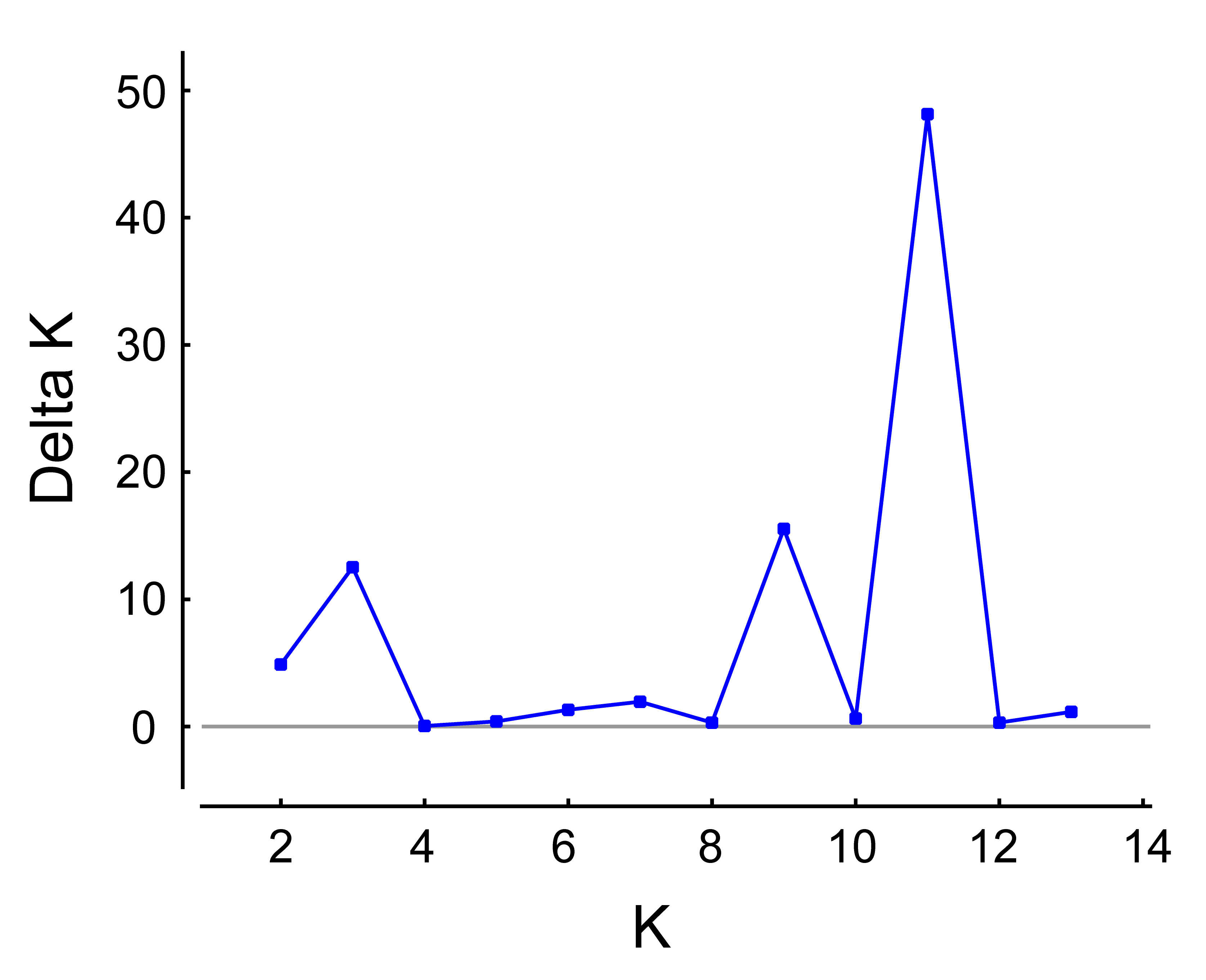

Supplement: Supplementary file 2 — Figure S1 [file ECE3-11-9498-s002.tif]

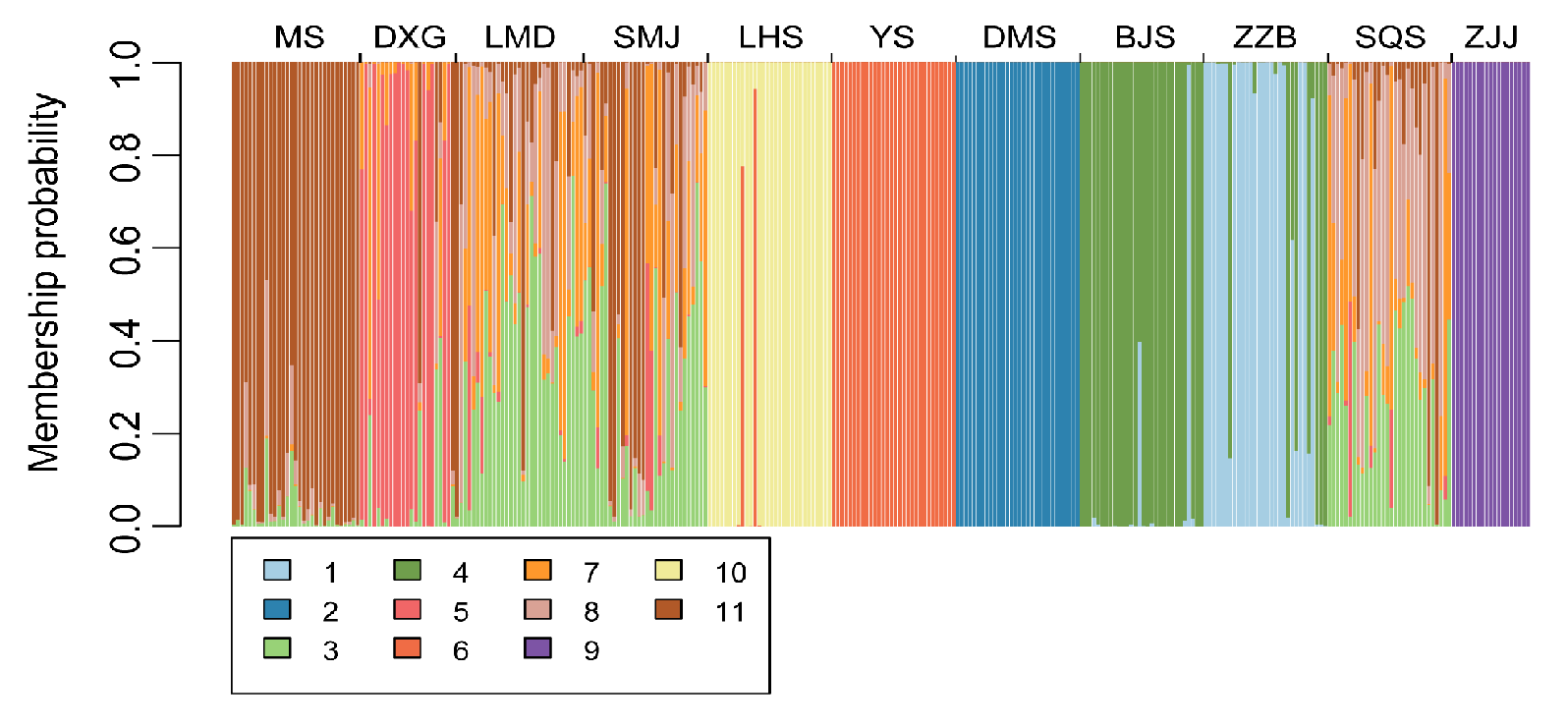

Supplement: Supplementary file 3 — Figure S2 [file ECE3-11-9498-s004.tif]

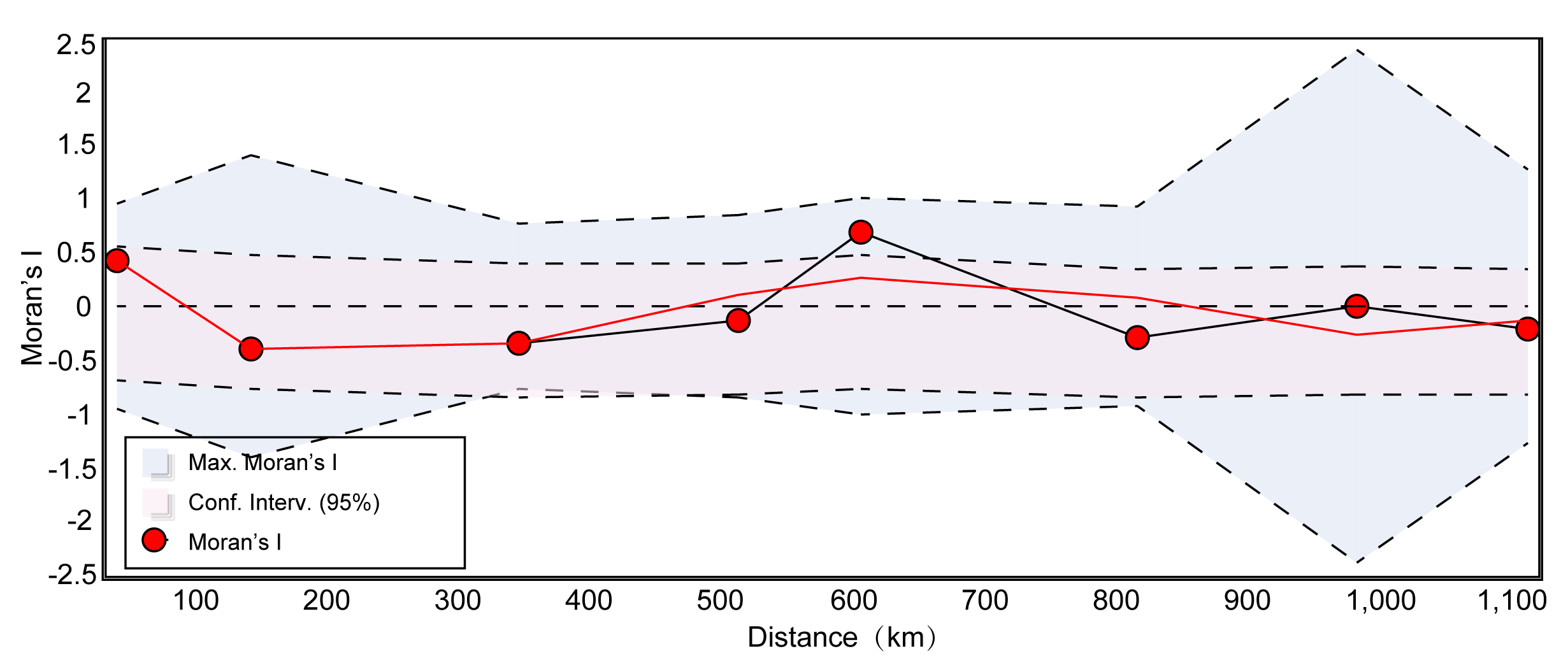

Supplement: Supplementary file 4 — Figure S3 [file ECE3-11-9498-s005.tif]

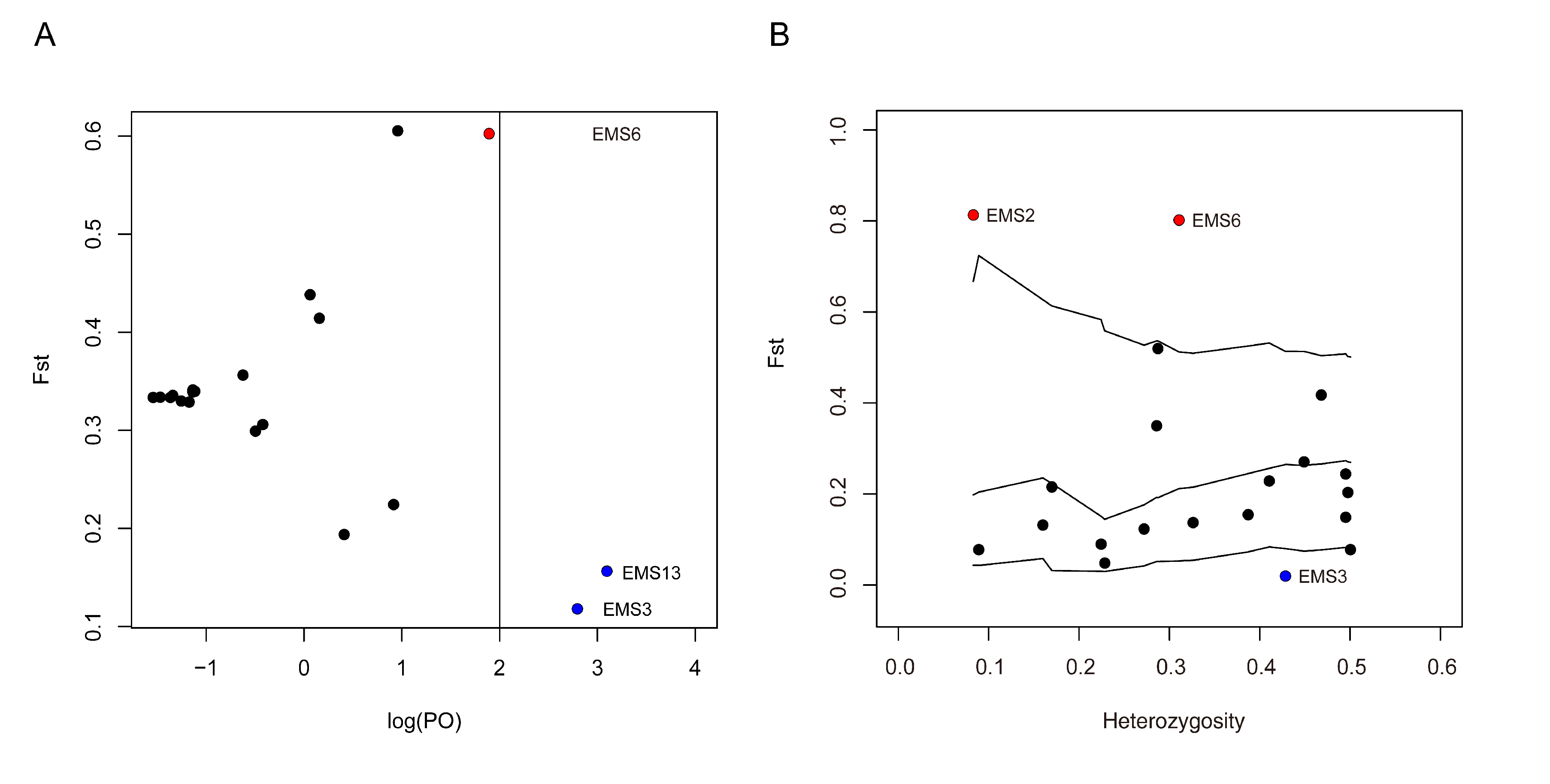

Supplement: Supplementary file 5 — Figure S4 [file ECE3-11-9498-s003.tif]
